# Spatiotemporal Analysis of Glucagon Secretory Granule Dynamics

**DOI:** 10.1111/tra.70019

**Published:** 2025-09-24

**Authors:** Samuele Ghignoli, Valentina De Lorenzi, Gianmarco Ferri, Licia Anna Pugliese, Marta Tesi, Piero Marchetti, Stefano Luin, Francesco Cardarelli

**Affiliations:** ^1^ NEST Laboratory Scuola Normale Superiore Pisa Italy; ^2^ Fondazione Pisana per la Scienza Pisa Italy; ^3^ Department of Clinical and Experimental Medicine, Islet Cell Laboratory University of Pisa Pisa Italy

**Keywords:** cytoskeleton, fluorescence, imaging‐derived mean square displacement, secretion, single particle tracking, α cells, β cells

## Abstract

The secretion of insulin and glucagon by pancreatic β and α cells, respectively, is critical for glucose homeostasis. While the insulin granule dynamics are well‐characterized, the intracellular behavior of glucagon secretory granules (GSG) remains poorly understood. Here, we analyze the mobility of GSGs in αTC1‐9 cells and insulin secretory granules (ISG) in INS‐1E cells using spatiotemporal correlation spectroscopy and single‐particle tracking (SPT), with a focus on the role of the cytoskeleton in regulating their transport. Under basal conditions, SPT classification reveals that GSGs predominantly exhibit diffusive motion (57.6% ± 10%), with smaller fractions categorized as almost immobile (35.8% ± 10.6%) or drifted (6.6% ± 3%), closely resembling ISG dynamics. By disrupting microtubules, we confirmed their role as active tracks for directed granule transport in both cell types. Upon exposure to their respective secretory stimuli—high glucose for β cells and low glucose for α cells—both granule populations underwent a comparable shift toward increased diffusive and drifted motions. Treatment with the actin depolymerizing agent Latrunculin‐B reproduced this stimulatory effect in INS‐1E cells but not in αTC1‐9 cells, suggesting that despite their overall similarity in granule behavior under physiological conditions, α and β cells may rely on partially distinct mechanisms to engage the cytoskeletal network.

## Graphical abstract

The schematic abstract illustrates the workflow of the study. Confocal image stacks were acquired from α cells (glucagon granules) and β cells (insulin granules) labeled with ZIGIR. Single‐particle tracking analysis was then performed to classify granule trajectories under various conditions. A custom‐made MATLAB script was used to categorize trajectories into blocked, diffusive, and drifted types. This initial dynamic analysis of glucagon granules reveals differences in their behavior compared to insulin granules, likely reflecting distinct secretory requirements.

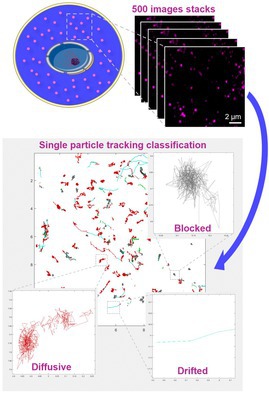

## 1 Introduction

Glucose homeostasis is tightly regulated by the opposing actions of insulin and glucagon, secreted by pancreatic β and α cells, respectively, within the islets of Langerhans [1, 2, 3]. These hormones are stored in secretory granules and released in response to metabolic cues, following a well‐coordinated process of biogenesis, maturation, intracellular trafficking, and exocytosis [4, 5, 6]. Understanding the intracellular dynamics of these granules is essential for deciphering the regulatory mechanisms that maintain glucose homeostasis and their dysfunction in metabolic diseases such as diabetes [7, 8]. While insulin granule dynamics in β cells have been extensively characterized, research on glucagon secretory granules (GSGs) in α cells remains limited. The process of insulin secretion begins with glucose metabolism, leading to ATP production, closure of ATP‐sensitive K^+^ channels, membrane depolarization, Ca^2+^ influx, and the mobilization of insulin‐containing granules toward the plasma membrane [5]. High‐resolution imaging techniques, including electron microscopy (EM) [9, 10], total internal reflection fluorescence (TIRF) microscopy [9, 11, 12, 13], spatiotemporal fluorescence correlation spectroscopy (FCS) [14], and single‐particle tracking (SPT) [15, 16, 17, 18], have provided a detailed picture of insulin secretory granules (ISGs) trafficking. These studies revealed that upon glucose stimulation, ISGs mobilization occurs in two phases: an initial rapid release of docked granules, followed by a sustained phase involving the transport of a reserve pool from deeper cytoplasmic regions [8, 19, 20]. Additionally, cytoskeletal elements were demonstrated to play distinct roles in regulating granule trafficking. Cortical F‐actin was demonstrated to act as a barrier that primarily restricts ISGs motion under basal conditions [15]; indeed, F‐actin extensive remodeling and/or depolymerization (e.g., induced by Latrunculin‐B) is thought to facilitate granule exocytosis upon glucose stimulation [21, 22, 23, 24]. Regarding α cells, limited data are available on the effect of F‐actin depolymerization on glucagon release as a pro‐secretory stimulus [25, 26]. On the other hand, microtubules were well demonstrated to serve as tracks for active ISGs transport, specifically to promote the sustained secondary phase of secretion [15]. For what concerns α cells, Yokawa and co‐workers measured reduced GSGs velocity upon NCZ‐induced microtubule depolymerization in α‐TC6 cells by spinning‐disk microscopy [27]. The limited understanding of GSGs dynamics must be attributed to both the historical focus on β cells (due to their central role in diabetes) as well as the limited availability of α cell models and suitable live cell labeling techniques for GSGs. To address these challenges, we utilized αTC1‐9 cells as an α cell model and INS‐1E and MIN6 cells as a β cell reference, and we exploited the ability of the fluorescent Zn^2+^ chelator ZIGIR to label these granule types [28]. In this work, we employed spatiotemporal FCS and single‐particle tracking (SPT) to characterize granule mobility under basal conditions, upon cytoskeletal perturbations, and in response to pro‐secretory stimuli. By comparing ISGs and GSGs trafficking, we aimed to identify shared and distinct regulatory mechanisms that could shed light on α‐cell physiology and its role in glucose homeostasis.

## 2 Results

### 2.1 Labeling of Glucagon and Insulin Granules by ZIGIR

**FIGURE 1:**
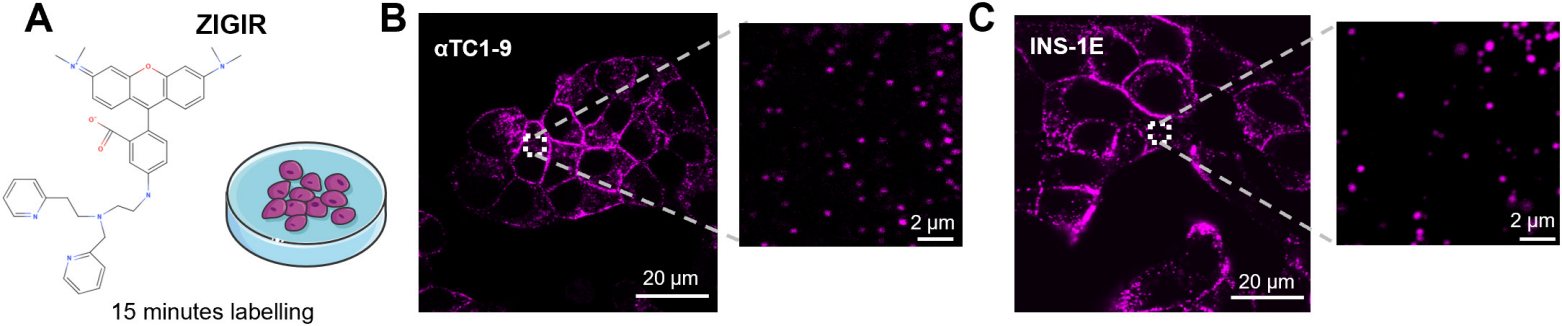
Granules labelling method. (A) Schematic representation of ZIGIR labelling protocol in which a single administration of ZIGIR 15 min prior to confocal microscopy imaging results in a punctate cellular labelling pattern. Servier Medical Art (https://smart.servier.com/) was used to create part of this figure. (B, C) Representative images of αTC1‐9 cells (B) and INS‐1E cells (C) in which GSGs and ISGs were labelled by ZIGIR.

To fluorescently label secretory granules in living αTC1‐9 and INS‐1E cells, we utilized a recently developed method based on the Zn^2+^‐chelator ZIGIR [28]. According to the manufacturer's protocol, the ZIGIR‐based labeling involves administering a single dose of 1 μM, waiting at least 15 min before live‐cell imaging (Figure 1A, see Section 4 for further details). Upon applying this protocol to our system, nearly all cells in the petri dish exhibited punctate fluorescent staining, predominantly near the plasma membrane, in both αTC1‐9 cells (Figure 1B) and INS‐1E cells (Figure 1C). As a reference, we used transfection of EGFP‐tagged neuropeptide Y (NPY‐EGFP) to label GSGs in αTC1‐9 cells (Figure S1) [29]. Notably, ZIGIR and NPY‐EGFP signals in live imaging overlapped in punctate structures throughout the cytoplasm. In addition, sparse NPY‐EGFP signal was detected, likely due to the presence of fully mature NPY‐EGFP proteins within the Golgi network (Figure S2A,B). Moreover, we confirmed that the labelled vesicles are indeed GSGs by performing anti‐glucagon immunofluorescence on NPY‐EGFP transfected αTC1‐9 cells (Figure S2C,D).

### 2.2 Validation of Live Cell Imaging Protocol via Spatiotemporal FCS

**FIGURE 2:**
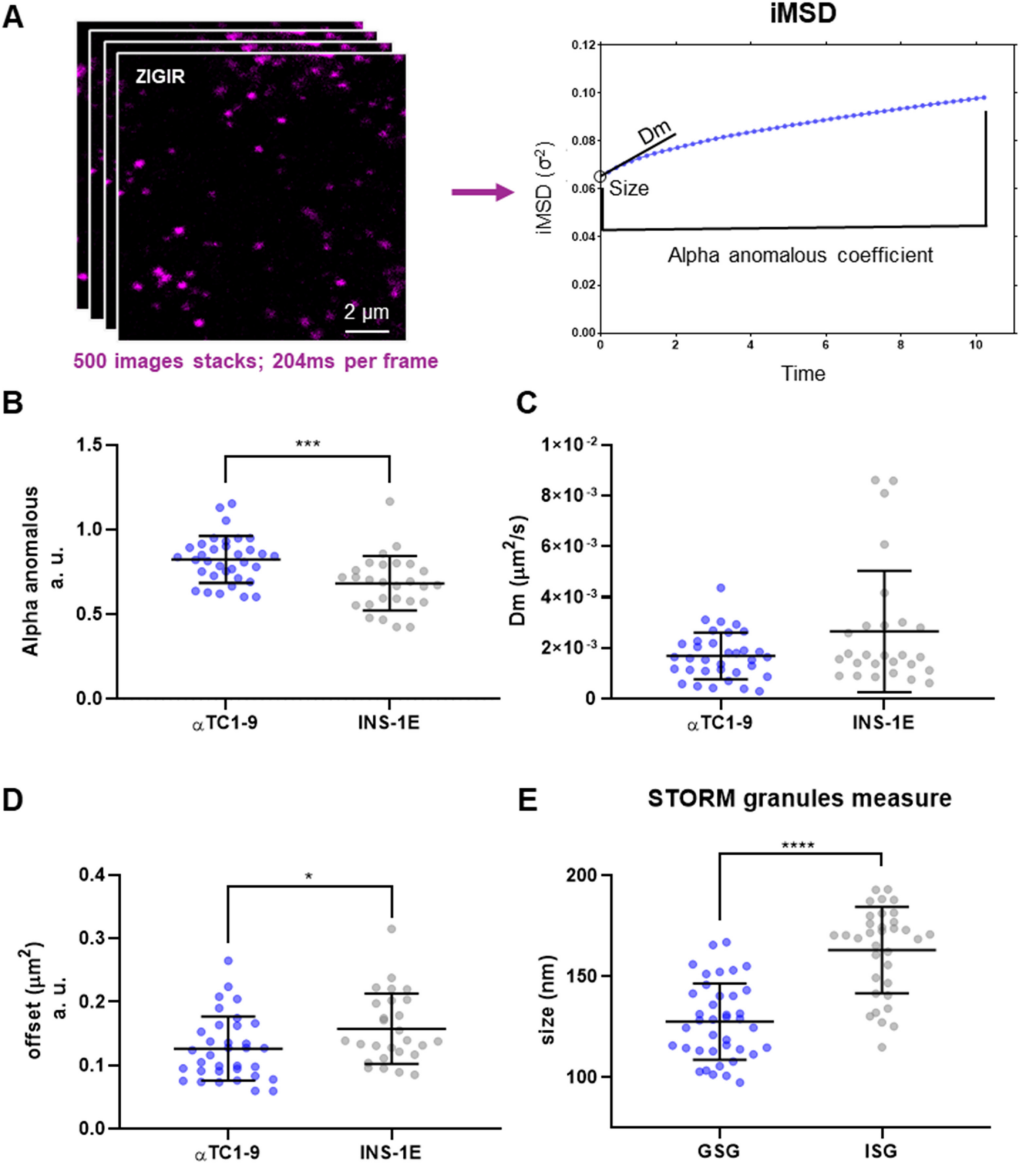
iMSD and STORM comparison between αTC1‐9 cells and INS‐1E cells. (A) 500‐frames movies of confocal images of labelled GSGs and ISGs in αTC1‐9 cells and INS‐1E cells, respectively, were acquired at ~200 ms per frame; the movies were processed by a custom made iMSD script for average dynamical and structural analysis. A summary graph of the results obtained by the iMSD script: The alpha anomalous coefficient, the diffusion parameter at short length scale *D*_*m*_ and the apparent size parameter (offset). See Section 4 for details. (B–D) Comparison of the alpha anomalous coefficient, *D*_*m*_, and offset (squared apparent size) obtained for αTC1‐9 cells (*N* = 34) and INS‐1E cells (*N* = 27) labelled by ZIGIR. Each dot shown in the scatter plots represents the outcome from a single acquisition; horizontal gray lines with error bars are mean ± SD. (B) In both cell types the motion of the granules was sub‐diffusive, even if the alpha anomalous coefficient for GSGs in αTC1‐9 cells (0.82 ± 0.14) was closer to 1 than for ISGs in INS‐1E cells (0.64 ± 0.14; mean ± SD). (C) No significant differences were ascertained for the diffusivity, despite some outliers in both cases but mostly in INS‐1E cells. αTC1‐9 cells *D*_*m*_ = 1.5 * 10^−3^ ± 0.9 * 10^−3^ μm^2^/s and INS‐1E cells *D*_*m*_ = 2.3 * 10^−3^ ± 1.9 * 10^−3^ μm^2^/s (mean ± SD). (D) A difference was present for the offset, highlighting a difference in apparent size, indicating that GSGs on average resulted smaller than ISGs. (E) Size results from STORM imaging of super‐resolved granules comparing GSGs diameter (132.5 ± 39.0 nm) and ISGs diameter (169.7 ± 56.4 nm). Each dot shown in the scatter plot represents a single granule measurement; horizontal gray lines with error bars are mean ± SD. We checked normality and performed t test for the normally distributed data and Mann–Whitney nonparametric test for the non‐normally distributed data.

To evaluate the time‐resolved live‐cell imaging protocol used to assess granule dynamics, we acquired confocal image stacks of 500 frames of ZIGIR‐labeled granules in living cells (i.e., ~5 images per second or ~200 ms temporal resolution). Then, we performed iMSD (imaging‐derived Mean Square Displacement) analysis on these image stacks (Figure 2A) [14, 30, 31]. iMSD, in fact, is a rapid, robust spatiotemporal correlation spectroscopy technique that provides a real‐time quantitative estimate of organelle diffusivity *D*_*m*_ (D *micro* represents the diffusivity at short time and space scales, reflecting the effective free diffusion that may occur within confined areas) and of the overall mode of motion (through the alpha anomalous coefficient), while simultaneously probing average granule size information through the offset (the *y*‐axis intercept) of the iMSD plot.

As extensively discussed elsewhere [14, 30, 31], this offset corresponds to the squared waist of the spatial correlation function at zero lag time (*σ*_0_^2^). This value incorporates contributions from both the squared width of the point spread function and the squared average particle size [14, 30, 31]. Consequently, the offset increases with granule dimension.

We compared ZIGIR‐labeled GSGs in αTC1‐9 cells with ISGs in INS‐1E cells (Figure 2B–D) through iMSD. A couple of observations can be made: first, ZIGIR proved to be a reliable marker for Zinc‐rich organelles, showing consistency in the dynamic properties of GSGs labeled with NPY‐EGFP [29] (Figure S3A), as well as ISGs labeled with C‐pep‐EGFP [14] (Figure S3B). Second, as expected, iMSD analysis shows that GSGs are principally diffusive, with average properties comparable to those of ISGs (see alpha anomalous parameter and *D*_*m*_ in Figure 2B,C); indeed, as discussed in [32], an average alpha anomalous coefficient slightly below 1 is commonly associated with predominantly diffusive motion within cells. This deviation from ideal Brownian diffusion reflects the presence of intracellular hindrances, such as membranes, organelles, or cytoskeleton elements, leading to slightly sub‐diffusive behavior. Statistical analysis of the offset values reveals a difference in size between GSGs and ISGs, with ISGs generally bigger than GSGs (Figure 2D). It is important to note that inadequate temporal resolution and the point spread function (PSF) width can lead to an overestimation of the granule sizes [30], so we decided to further validate this difference by direct measurements of granule size in both cell types using Stochastic Optical Reconstruction Microscopy (STORM), as reported in Figures 2E and S4. The results confirmed the trend observed by the offset in the iMSD and are consistent with previous dense core measurements performed by transmission electron microscopy (TEM) and scanning electron microscopy (SEM) [10, 33, 34].

### 2.3 SPT‐Based Classification of Granule Dynamics Under Upkeeping Conditions

**FIGURE 3:**
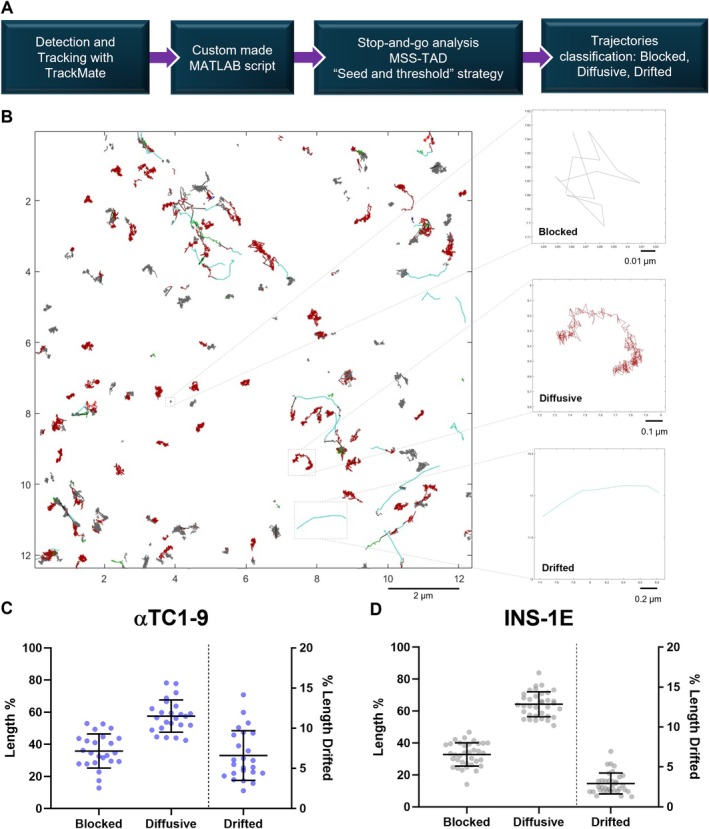
SPT analysis for particle trajectories classification of αTC1‐9 cells and INS‐1E cells in upkeeping conditions. (A) Summary scheme of the principal steps of the analysis for the trajectory segmentation and classification, leading to the final division into blocked, diffusive and drifted (sub)trajectories. (B) Representative output of the custom‐made MATLAB script, highlighting the results of segmenting the trajectories and the classification into blocked, diffusive and drifted parts (for details see material and methods): Gray (sub)trajectories are blocked, red (sub)trajectories are diffusive and both cyan and green ones are drifted (from the first and second step of the analysis, respectively); black segments are connections between different sub‐trajectories within the same trajectory. (C‐D) Resulting fraction of granules undergoing the specified motion mode for different αTC1‐9 cells (*N* = 24) (C) or INS‐1E cells (*N* = 35) (D) in upkeeping conditions. The graph represents the percentage (%) of the total length of sub‐trajectories classified in the three different modes. “Drifted” y‐axis on the right (% Length Drifted). Scatter plots with mean ± SD with each dot representing the outcome from a single acquisition.

The intrinsic heterogeneity of dynamic parameters within the population of diffusing granules is inevitably averaged out by the iMSD algorithm. Additionally, iMSD analysis is primarily sensitive to the mobile, diffusing population of granules, while it tends to underrepresent or overlook stationary granules [35, 36, 37]. With iMSD, granules with varying mobilities and diffusion characteristics are analyzed collectively along with stationary granules, or those with minimal displacement, which mitigates the underlying differences in their dynamic parameters. To capture all relevant information, we employed single‐particle tracking (SPT) analysis using the TrackMate plugin for ImageJ to construct the tracks and custom MATLAB scripts to analyze them (Figure 3A, see Section 4 for further details). Briefly, we used TrackMate for particle recognition, spatial localization, and trajectory reconstruction [38, 39]; granules were detected using a Laplacian of Gaussians (LoG) filter, with an estimated object diameter of 0.3 μm, consistent with our observations, considering PSF deformation due to confocal imaging (Figure 2D,E). The tracking algorithm was based on a linear assignment problem (LAP) [40], without accounting for splitting or merging events. Following TrackMate analysis, a custom MATLAB script was used for data analysis and trajectory classification (see Section 4) [37]. This approach enabled us to separate the trajectories of the granules into sub‐trajectories characterized by a single motion mode, when needed, and to classify them into three categories: “blocked,” “diffusive,” and “drifted.” The “blocked” category represents immobile or strongly confined granules, the “diffusive” category pertains to granules diffusing freely, possibly within a confined region, and the “drifted” category includes granules moving in fast, directed steps (Figure 3B). In brief, starting from trajectories obtained from the TrackMate analysis, the first step of classification looks for (parts of) trajectories characterized by a fast directed motion, in a sort of “stop‐and‐go” analysis [41, 42, 43]. The remaining (sub)trajectories (meaning both trajectories and sub‐trajectories) are often still multimodal, therefore in the second step of classification they are segmented looking for “transient arrest of diffusion” (TAD) zones. The resulting (sub)trajectories are classified according to the type of motion through MSS‐TAD analysis, which differentiates blocked or slow (sub)trajectories from diffusive or fast ones [32, 37]. The drifted sub‐trajectories resulting from this second step of the classification are merged with the faster drifted ones obtained previously. Classification criteria have been optimized through parameter tests (see Section 4). A key parameter was the threshold velocity, *v*_*t*_, set at 0.2 μm/s, which defined the lower velocity limit for classifying particles as drifted in the first step of analysis. This threshold value proved to be optimal for separating drifted trajectories from diffusive and/or confined ones, highlighting the distinction between fast/directed and slow/confined movement.

This SPT approach enabled us to classify the dynamics of both GSGs and ISGs in upkeeping condition (Figure 3C,D, respectively). We defined the upkeeping condition as standard cell culture maintenance condition, without external stimuli or perturbation. The SPT classification of GSGs in αTC1‐9 cells revealed a pattern in which diffusive behavior dominates (57.6% ± 10%), over blocked (35.8% ± 10.6%) and drifted ones (6.6% ± 3%, Figure 3C, Table 1). These percentages represent the proportion of granules exhibiting each type of motion relative to the total number of granules tracked across all trajectories and frames, effectively quantifying the fraction of time that the granules spend in a motion category. Noteworthy, GSGs exhibited a notably higher propensity for directed motion compared to ISGs (6.6% vs. 2.9%, Table 1), a trend also observed through iMSD analysis of the entire mobile granule population (Figure 2B), despite the lesser sensibility of this technique. Notably, the classification results for ISGs in INS‐1E cells under upkeeping conditions closely aligned with findings from other studies using similar SPT‐based approaches [9, 15], showing a clear predominance of diffusive behavior (64.3% ± 7.7%) over blocked (32.8% ± 7.2%) and drifted (2.9% ± 1.3%) ones (Figure 3D, Table 1). To characterize motion dynamics, we computed the joint distribution of the short‐lag‐time diffusion coefficient (*D*_12_, corresponding to *D*_*m*_ but for each (sub)trajectory) and the anomalous diffusion parameter *γ* [32], which is functionally analogous to (half of) the alpha anomalous coefficient obtained from iMSD analysis. Therefore, it provides equivalent insight into dynamic properties (Figure S5). In αTC1‐9 cells under upkeeping conditions, the resulting graph shows a peak slightly under *γ* = 0.5, indicating a motion very close to Brownian but with a tendency of sub‐diffusive behavior (Figure S5A). In INS‐1E cells under upkeeping condition, the peak appears quite below *γ* = 0.5, indicating a more strongly sub‐diffusive motion (Figure S5B). In summary, *γ* analysis from SPT script confirms the findings of the iMSD‐derived alpha anomalous coefficient; the similarity between αTC1‐9 and INS‐1E cells for the distributions of *D*_12_ values, and for their peaks, reflects the lack of significant differences among the *D*_*m*_ population as shown in Figure 2C.

**TABLE 1:** Summary table of the classified categories for different conditions.

Cell model and conditions	Blocked	Diffusive	Drifted	*N*
αTC1‐9	35.8 ± 10.6	57.6 ± 10	6.6 ± 3	24
INS‐1E	32.8 ± 7.2	64.3 ± 7.7	2.9 ± 1.3	35
αTC1‐9 NCZ	56.1 ± 10.7	40.4 ± 10.2	3.5 ± 1.7	17
INS‐1E NCZ	48.2 ± 9.8	50.1 ± 9.8	1.7 ± 0.6	17
αTC1‐9 Lat‐B	58.3 ± 7.2	33.6 ± 8.1	8.1 ± 4.5	17
INS‐1E Lat‐B	19.2 ± 6.4	71.0 ± 6.9	9.8 ± 3.1	16
αTC1‐9 ctrl Hypo (2.5 mM glucose)	28.6 ± 7.3	65.9 ± 7.3	5.5 ± 2	17
INS‐1E ctrl High Gluc (2.5 mM glucose)	44.8 ± 10.6	53.1 ± 10.5	2.1 ± 0.9	22
αTC1‐9 Hypo (1 mM glucose)	19.6 ± 5.2	72.1 ± 5	8.3 ± 3.6	19
INS‐1E High Gluc (16.7 mM glucose)	35.1 ± 9.2	61.5 ± 9.3	3.4 ± 2	25
αTC1‐9 ctrl High Gluc (2.5 mM glucose)	28.2 ± 5.7	67.2 ± 5.3	4.6 ± 2.2	23
INS‐1E ctrl Hypo (2.5 mM glucose)	42.4 ± 11.1	55.1 ± 10.5	2.5 ± 1.8	31
αTC1‐9 High Gluc (16.7 mM glucose)	28.4 ± 6.3	67.1 ± 6.5	4.5 ± 1.8	24
INS‐1E Hypo (1 mM glucose)	39.5 ± 9.8	58.4 ± 9.4	2.1 ± 1.7	35

*Note:* To better read the table, αTC1‐9 cells and INS‐1E cells are alternated with the first on gray rows and the seconds on white rows. *N* is the numerosity of samples and all the data are expressed as length percentage with mean ± SD.

Abbreviations: High Gluc, high glucose stimulus; Hypo, hypoglycemia condition; Lat‐B, Latrunculin‐B; NCZ, nocodazole.

### 2.4 SPT‐Based Classification of Granule Dynamics: The Role of Cytoskeleton Components

**FIGURE 4:**
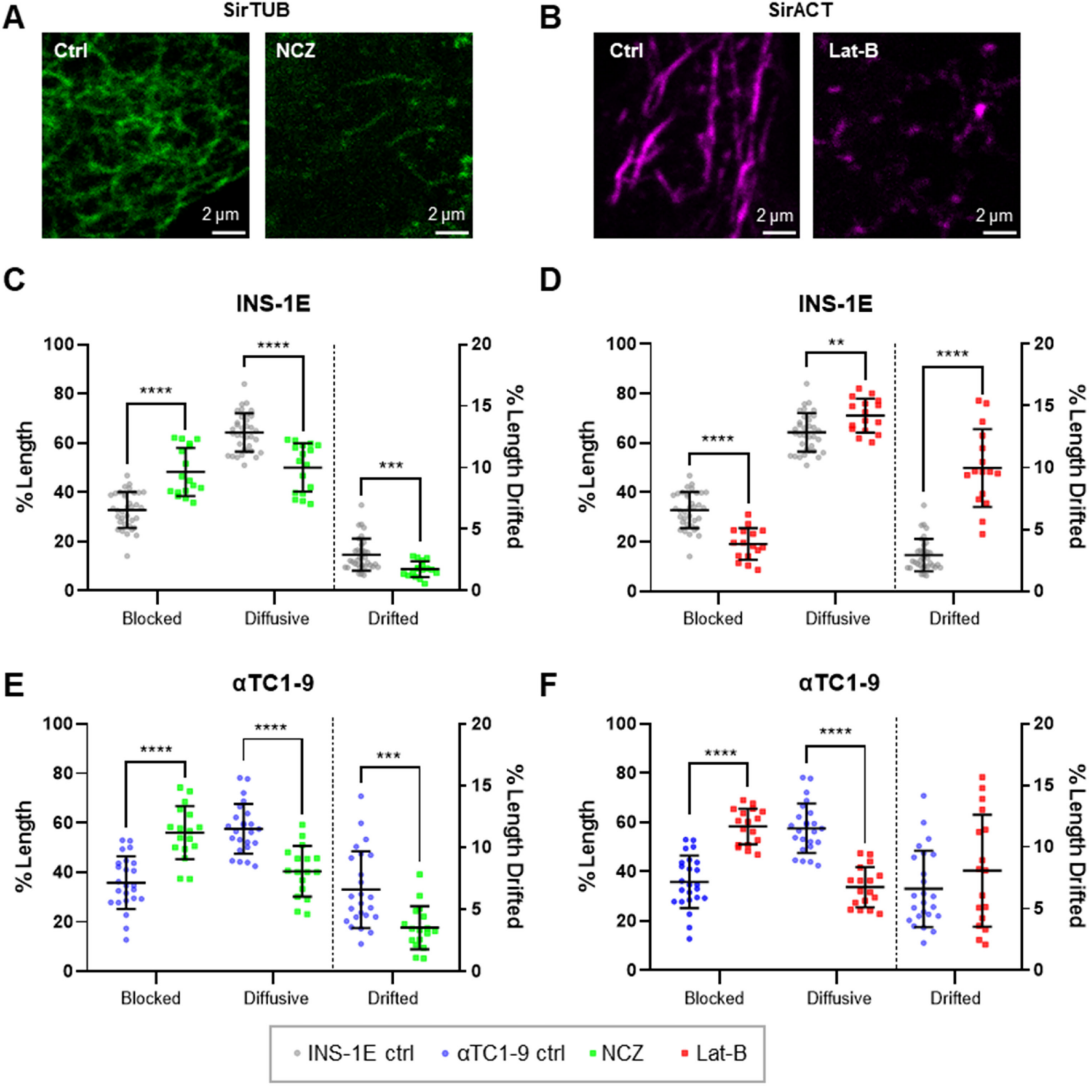
SPT classification for ISGs and GSGs trajectories after Nocodazole (NCZ) and Latrunculin‐B (Lat‐B) treatment. (A) Tubulin is labelled with sirTubulin (SirTUB) in green in control condition (Ctrl) and after NCZ treatment, in which the cytoskeleton appears fragmented. (B) Actin is labelled with sirActin (SirACT) in violet in control condition (Ctrl) and after Lat‐B treatment, in which the Actin filaments appear fragmented. (C–F) Box plots for 3 categories of granules trajectories in which we compare the upkeeping condition (ctrl) with respect to cytoskeletal component depolymerization. (C) Comparison between ISGs ctrl condition (*n* = 35, same data of Figure 3D) and microtubule depolymerization (NCZ, *n* = 17). NCZ treatment caused an increase in blocked trajectories to the detriment of both diffusive and drifted ones, denoting a reduction of general motility. “Drifted” *y*‐axis on the right (% Length Drifted). (D) Comparison between ISGs ctrl condition (*n* = 35, same data of Figure 3D) and disrupted Actin filaments one (Lat‐B, *n* = 16). Lat‐B induced a net decrease in blocked trajectories and raised up both diffusive and drifted ones. “Drifted” y‐axis on the right (% Length Drifted). (E) Comparison between GSGs ctrl condition (*n* = 24, same data of Figure 3C) and microtubules disruption one (NCZ, *n* = 17). Here the treatment increased the blocked trajectories with a concomitant decrease of the motility fraction of the trajectories. “Drifted” *y*‐axis on the right (% Length Drifted). (F) Comparison between ctrl condition (*n* = 24, same data of Figure 3C) and disrupted Actin filaments one (Lat‐B, *n* = 17). The result is almost the opposite of the one for ISGs, with an increase of blocked trajectories and a concomitant decrease in diffusive trajectories. “Drifted” *y*‐axis on the right (% Length Drifted). We checked normality and we performed multiple t test for the normally distributed data using Holm–Sidak correction method (alpha = 0.05); each row of data was analyzed individually, without assuming a consistent SD. Scatter plots with mean ± standard deviation with each dot shown represents the outcome from a single acquisition.

To evaluate the influence of cytoskeleton components on granule dynamics in upkeeping condition, we performed SPT analysis in the presence of cytoskeleton‐disrupting agents, that is, 10 μM Nocodazole (NCZ) to induce microtubule depolymerization and 10 μM Latrunculin‐B (Lat‐B) to induce F‐actin depolymerization (Figure 4A,B). As mentioned in the introduction, previous studies have well characterized the role of cytoskeleton in β cells: microtubules serve as tracks for active granule transport, especially supporting the sustained secondary phase of secretion [15], while cortical F‐actin primarily acts as a barrier to ISG motion under basal conditions, and it is subjected to depolymerization to facilitate exocytosis upon glucose stimulation [21, 22, 23]. In line with these expectations, NCZ treatment in INS‐1E cells produced a significant increase in the proportion of ISGs exhibiting blocked trajectories compared to control condition, at the expense of mobile trajectories (See Table 1). Notably, the drifted trajectories decreased (from 2.9% ± 1.3% to 1.7% ± 0.6%, mean ± SD, Figures 4C and S6A,B, Table 1). INS‐1E cells responded as expected to the pro‐secretory stimulus of Lat‐B‐induced F‐actin depolymerization, showing substantial reduction of the fraction of blocked ISG trajectories and a corresponding increase in drifted‐motion trajectories (Figures 4D and S6A–C, Table 1). These changes in dynamics are evident also upon iMSD analysis: we observed a significant increase in *D*_*m*_, from 3.35 * 10^−3^ μm^2^/s to 12.8 * 10^−3^ μm^2^/s (Figure S7A). Considering GSG dynamics, the αTC1‐9 cells responded to NCZ‐induced microtubule depolymerization similarly to INS‐1E cells, with a neat increase in the fraction of blocked granules and a concomitant reduction in both diffusive and drifted categories (Figures 4E and S6D,E, Table 1). Their response to F‐actin depolymerization, instead, did not match the results from INS‐1E cells. Indeed, the fraction of immobile GSGs increased significantly compared to the control (blocked from 35.8% ± 10.6% to 58.3% ± 7.2%, mean ± SD, Figures 4F and S6D–F, Table 1), mostly at the expense of diffusive trajectories (from 57.6% ± 10% to 33.6% ± 8.1%, mean ± SD, Figures 4F and S6D–F, Table 1), with no significant shift in the fraction of drifted ones. This kind of behavior could also be highlighted with iMSD by analyzing the alpha anomalous coefficient, whose average decreased from 0.82 for untreated cells to 0.45 for Lat‐B‐treated cells (Figure S7B), highlighting a very sub‐diffusive motion in this last case. INS‐1E cells and αTC1‐9 cells employed in this comparison derived from rat and mouse, respectively. To exclude that the observed difference derives from species specificities, we employed MIN6 mouse cells as β cell model to pair the results directly with mouse αTC1‐9 cells. We performed SPT on ISGs of MIN6 after cytoskeletal alterations obtaining the same behavior of INS‐1E β cells. In detail, NCZ reduced the mobility by increasing the blocked trajectories (from 47.8% ± 12.2% to 59.3% ± 12.7%, mean ± SD, Figure S8A, Table S2) and decreasing both diffusive (from 45.4% ± 11.1% to 36.3% ± 10.9%, mean ± SD, Figure S8A, Table S2) and drifted ones (from 6.8% ± 3.4% to 4.4% ± 3.3%, mean ± SD, Figure S8A, Table S2). Lat‐B, instead, increases the mobility of these granules as happened for INS‐1E β cells, by decreasing blocked trajectories (from 47.8% ± 12.2% to 27.7% ± 10.1%, mean ± SD, Figure S8B, Table S2) and increasing both diffusive (from 45.4% ± 11.1% to 51.1% ± 10.5%, mean ± SD, Figure S8B, Table S2) and drifted ones (from 6.8% ± 3.4% to 21.2% ± 6.9%, mean ± SD, Figure S8B, Table S2). Notably, the dispersion of the data is higher compared to INS‐1E denoting a broad variability and heterogeneity in this cell line [44].

### 2.5 SPT‐Based Classification of Granule Dynamics Under Secretory Stimuli

**FIGURE 5:**
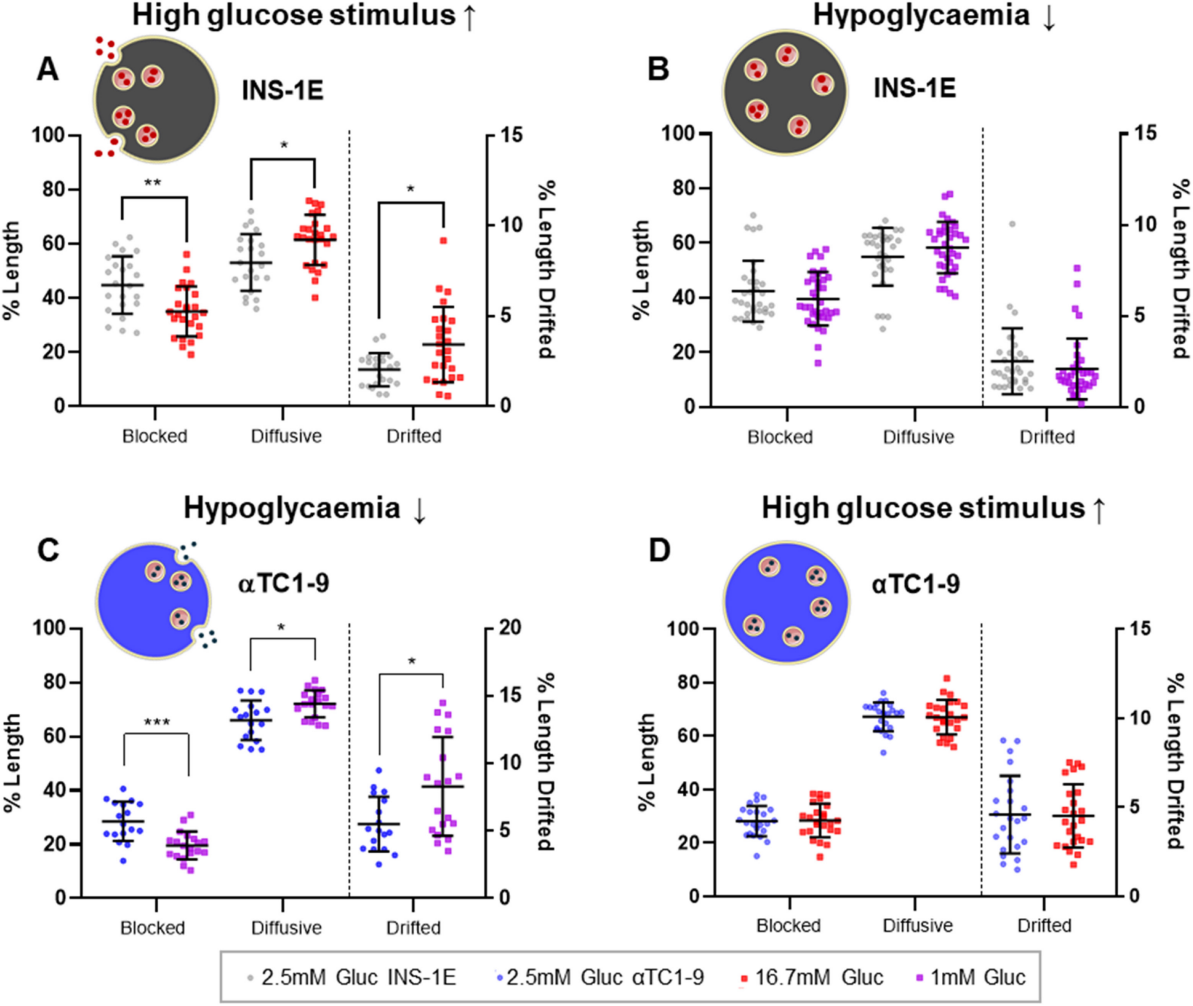
Alteration of granules trajectories after secretory stimuli (High Glucose) for INS‐1E cells and after hypoglycaemia stimulus for αTC1‐9 cells, and the related counterpart. Scatter plots showing the three categories (blocked, diffusive, and drifted) of GSGs and ISGs motion modes with a schematic representation of cells during the event of secretion or with no secretion. We collected the images within 1 h from the stimulus to check the motility alteration. “Drifted” y‐axis on the right (% Length Drifted) for each graph. (A) ISGs motility after high glucose stimulus. Comparison between the control condition in KRBH buffer (2.5 mM Gluc, *n* = 22) with respect to secretory stimulus of high glucose (16.7 mM Gluc, *n* = 25). (B) ISGs motility in hypoglycaemia condition. Comparison between control condition in KRBH buffer (2.5 mM Gluc, *n* = 31) and hypoglycemia treatment (1 mM Gluc, *n* = 35) showing no alteration in any category of trajectories, as expected. (C) GSGs motility in hypoglycaemia condition. Comparison between control condition in KRBH buffer (2.5 mM Gluc, *n* = 17) and hypoglycemia treatment (1 mM Gluc, *n* = 19). Hypoglycemia treatment induces a general increase in motility by directly decreasing the blocked trajectories and raising up both diffusive and drifted, as happens in the case of high glucose stimuli for INS‐1E cells. We collected the images within 1 h starting from the treatment with 1 mM glucose media. (D) GSGs motility in αTC1‐9 cells after high glucose stimulus. We perform the same glucose stimulus used for the data in panel A, confirming the absence of alteration in motility (2.5 mM Gluc, *n* = 23; 16.7 mM Gluc, *n* = 24). We checked normality and performed multiple t tests for the normally distributed data using the Holm‐Sidak correction method (alpha = 0.05); each row of data was analyzed individually, without assuming a consistent SD. Servier Medical Art (https://smart.servier.com/) was used to create part of this figure. Scatter plots with mean ± SD with each dot shown represent the outcome from a single acquisition.

The discordant behavior of αTC1‐9 and both INS‐1E and MIN6 cells in response to Lat‐B‐induced F‐actin depolymerization, a putative pro‐secretory stimulus for both cell types [24, 25, 26, 45], prompted us to evaluate whether the same difference persisted under their respective secretory stimuli induced by glucose concentration. Thus, we investigated αTC1‐9 and INS‐1E cells responses to low (hereafter hypoglycemia) and high glucose concentration, respectively. The analyzed granule population includes both pre‐existing and newly produced granules following stimulation [9, 29, 46], and we examined how their mobility changed under these conditions. In INS‐1E cells, exposure to its standard secretory stimulus (increasing glucose concentration from 2.5 to 16.7 mM) resulted in a clear decrease in the fraction of immobile ISGs (from 44.8% ± 10.6% to 35.1% ± 9.2%, Figure 5A, Table 1), accompanied by an increase in mobile granules, particularly those exhibiting drifted motion (from 2.1% ± 0.9% to 3.4% ± 2.0%, Figure 5A, Table 1), consistent with previous findings [15] and with the pro‐secretory effect observed using Lat‐B. As a control, INS‐1E cells subjected to hypoglycemic conditions exhibited no changes in ISGs dynamics (Figure 5B, Table 1). To assess GSGs mobility under secretory stimulation, the αTC1‐9 cells were incubated in KRBH supplemented with 1 mM glucose after a 45–60 min preincubation period in KRBH supplemented with 2.5 mM glucose, as previously reported to simulate hypoglycemia in this α cells model [47] (see Section 4). Image acquisition was completed within 30–40 min from the start of incubation. Exposing the αTC1‐9 cells to the hypoglycemic stimulus led to a marked reduction in the proportion of blocked GSGs (from 28.6% ± 7.3% to 19.6% ± 5.2%, Figure 5C, Table 1), accompanied by a clear increase in the population of motile granules (from 65.9% ± 7.3% to 72.1% ± 5% for diffusive granules, and from 5.5% ± 2% to 8.3% ± 3.6% for drifted ones, Figure 5C, Table 1). On the contrary, increasing glucose concentration (up to 16.7 mM) on αTC1‐9 cells had no detectable effect on GSGs dynamics (Figure 5D, Table 1). SPT‐based classification of granule motion under secretory stimulation over time provided additional insights into temporal patterns. In INS‐1E β cells (Figure S9A), high glucose treatment induced an early rise in diffusive motion with a concomitant decrease in blocked fraction, both of which gradually returned to baseline, while the drifted population showed a transient peak at 10–20 min likely indicating directed transport sustaining secretion. In αTC1‐9 cells (Figure S9B), hypoglycemia increased both diffusive and drifted populations while the blocked fraction stayed below control. Compared to INS‐1E, αTC1‐9 granules showed higher, longer‐lasting motility and greater variability in the drifted fraction. ELISA‐based quantification of glucagon in cell supernatants confirmed a consistent release at 1 mM glucose (Figure S10A), and a marked stimulation of secretion in response to Lat‐B, irrespective of glucose concentration (Figure S10B). Taken together, these findings indicate that, under their respective secretory stimuli, both αTC1‐9 and INS‐1E cells display a comparable increase in drifted and diffusive motions. This effect is phenocopied by Lat‐B treatment in INS‐1E cells (and MIN6 cells), but not in αTC1‐9 cells, pointing to distinct regulatory mechanisms governing their interactions with the cytoskeletal network.

## 3 Discussion

The intracellular trafficking of secretory granules is a key determinant of hormone release, yet while insulin granules have been extensively studied, glucagon granules remain poorly characterized. Our study was designed to establish a baseline comparative characterization of granule dynamics. Here, we provide a quantitative analysis of GSGs mobility in αTC1‐9 cells using a combination of advanced time‐resolved fluorescence microscopy techniques. As an initial step, we aimed to achieve a reliable labeling of GSGs in our α‐cell model to enable the analysis of their dynamics. For this purpose, we employed ZIGIR, a Zn^2+^‐chelating fluorescent probe previously validated as a selective marker for rich zinc granules by Zadeh and colleagues [28]. A key advantage of using ZIGIR is its ability to label both GSGs and ISGs in the same manner, making it suitable for direct comparison between αTC1‐9 and INS‐1E cells. In our system, ZIGIR provided efficient and consistent visualization and tracking of both granule populations, establishing a robust approach for studying their intracellular dynamics. Subsequently, we applied a fast and reliable spatiotemporal correlation spectroscopy approach (iMSD) to assess the nature of granule motion through the determination of the apparent diffusion coefficient (D_m_) and the anomalous diffusion coefficient (alpha anomalous). This analysis revealed that both granule populations predominantly exhibit sub‐diffusive behavior. Furthermore, iMSD analysis indicated differences in granule size, which we then quantified using dSTORM imaging, yielding mean diameters of approximately 132.5 ± 39.0 nm for GSGs and 169.7 ± 56.4 nm for ISGs. The reported diameters are in line with the dense‐core sizes measured in previous studies, by means of TEM [10, 33], FIB/SEM [34], and cryo‐TEM [34], both in immortalized cell lines and isolated islets. Since our measurements were obtained using dSTORM—a super‐resolution optical technique based on antibody labeling—we cannot determine whether the signal includes the low‐electron‐density halo typically seen by EM. However, the close correspondence with published dense‐core values might suggest that we are primarily detecting the dense‐core region. Worth mentioning, granule size estimates reported in the literature show substantial variability, which is likely attributable to a combination of biological differences, methodological approaches, and fixation protocols [10, 33, 34, 48, 49].

In addressing the core objective, spatiotemporal FCS allowed us to extract the average dynamic properties of GSGs, while a custom MATLAB‐based SPT approach enabled high‐resolution (~200 ms/frame) tracking of individual granules, classifying them into “blocked” (immobile), “diffusive” (random motion), and “drifted” (directed transport) categories. The SPT classification allowed us to resolve distinct motion categories, revealing that under basal conditions, GSGs predominantly exhibit diffusive motion (57.6% ± 10%), with smaller fractions categorized as almost immobile (35.8% ± 10.6%) or drifted (6.6% ± 3%). Interestingly, these distributions closely mirror those of ISGs, suggesting that the two granule types may share similar trafficking strategies in upkeeping conditions. The classification results for ISGs in INS‐1E cells are consistent with previous studies using similar SPT‐based approaches [9, 15]. We then explored the role of cytoskeletal components in regulating granule motion. Microtubule disruption significantly reduced directed motion for both granule types, potentially extending to αTC1‐9 cells the same conclusions on microtubule role as active tracks for long‐range granule transport already characterized in β cells [15, 50]. Worthy of mention, this is consistent also with previous observations in α cells: Yokawa and co‐workers measured reduced GSGs velocity upon NCZ‐induced microtubule depolymerization in α‐TC6 cells by spinning‐disk microscopy [27]. On the other hand, Lat‐B‐induced actin depolymerization, thought to act as a pro‐secretory stimulus in both cell types [24, 25, 26, 45], produced apparently contrasting effects: ISGs showed increased drifted motion and overall enhanced mobility, while GSGs exhibited no significant increase in directed motion, with an overall decrease in mobility. In addition, when the cells were challenged with their respective physiological secretory stimuli—high glucose for β cells and low glucose for α cells—both granule populations responded in a comparable manner, showing a parallel increase in diffusive and drifted trajectories. Our findings suggest that, while both cell types utilize the cytoskeleton for granule trafficking, their reliance on specific cytoskeletal components may differ, likely reflecting distinct regulatory mechanisms tailored to their secretory demands. Notably, previous studies have reported differences in the kinetics of exocytosis between human α and β cells. Although both cell types have been proven to display biphasic hormone secretion, as measured by TIRF microscopy, α cells exhibit markedly reduced exocytosis rates as compared to β cells [29]. As emerged from our work, the difference in secretory dynamics could be linked to the distinct granule motion patterns observed here, potentially reflecting adaptations to their respective physiological roles.

While the αTC1‐9 and INS‐1E cell lines provide practical and reproducible models to study α and β cell biology respectively, they have some limitations compared to primary islet cells such as partial glucose responsiveness, differences in gene expression profiles, and loss of intercellular paracrine interactions [51, 52]. However, the use of cell lines allowed us to solidly explore the details of glucagon granule dynamics, avoiding the morphological and functional heterogeneity of primary cells [53, 54, 55] providing a reference framework for comparing granule dynamics under different conditions. It should be noted that our study does not directly address the fate of the different granule populations in α cells. In this regard, drifted trajectories can be interpreted as signatures of kinesin‐driven transport along microtubules, likely sustaining secretion once the initial pool is depleted [15, 56]. By contrast, blocked granules may represent vesicles immobilized at cortical sites, either docked at the plasma membrane or retained by actin‐associated tethers [57, 58], while the diffusive fraction may not simply reflect passive Brownian motion but rather a combination of ATP‐dependent processes and cytoskeletal constraints [15]. Overall, these categories may represent interconnected states through which granules transit before becoming release competent. Investigating how specific motion patterns correlate with GSGs exocytosis remains an open and functionally relevant question. We consider this a natural direction for future studies, particularly in more physiological models such as intact pancreatic islets. Applying single particle tracking and granule dynamics analysis to human islets would provide a more integrated context for investigating cell‐type‐specific granule behavior. This model preserves the native three‐dimensional cytoarchitecture of the tissue as well as the natural interaction between α and β cells, thereby offering insights under more representative biological conditions. Regarding this, recent studies have demonstrated the feasibility of the specific labeling of ISGs and GSGs within intact islets [29, 59].

Through this work, we explored the dynamics of glucagon granules, revealing novel biologically relevant features of their interaction with the cytoskeletal network and uncovering unexpected differences from the closely related β cells. Further studies are needed to dissect the molecular basis of these differences, to understand why the α cells sometimes respond differently than the β cells, and to determine whether they contribute to dysregulated hormone secretion in metabolic disorders such as diabetes. By providing a comparative framework for studying ISG and GSG mobility, our findings lay the groundwork for future investigations into α cell physiology and its role in glucose homeostasis.

## 4 Materials and Methods

### 4.1 Cell Culture

Alpha TC1 clone 9 (αTC1‐9) cells (ATCC, cat# CRL‐2350, RRID: CVCL_0150) were cultured at 37°C with 10% CO_2_ in a controlled environment. They were maintained in DMEM low‐glucose medium (1 g/L = 5.5 mM) (Gibco, cat# 11880) supplemented with 10% heat‐inactivated fetal bovine serum (FBS), 15 mM HEPES, 2 mM L‐glutamine, 100 U/mL penicillin–streptomycin, 0.1 mM non‐essential amino acids (NEAA), and 0.02% bovine serum albumin (BSA). For β‐cell‐like cultures, we used INS‐1E cells, kindly provided by Prof. C. Wollheim (University of Geneva, Medical Center, RRID: CVCL_0351) and MIN6 mouse insulinoma cell line (SCC623). INS‐1E cells were grown in RPMI 1640 medium (Gibco, cat# 11835) containing 11.1 mM D‐glucose, supplemented with 10 mM HEPES, 100 U/mL penicillin–streptomycin, 1 mM sodium pyruvate, 2 mM L‐glutamine, 10% heat‐inactivated FBS, and 50 μM tissue culture‐grade β‐mercaptoethanol. They were maintained at 37°C with 5% CO_2_ in a controlled environment. These cell culture mediums were used in upkeeping conditions and for cytoskeleton‐disrupting experiments. MIN6 cells were grown in DMEM high‐glucose medium (4.5 g/L = 25 mM) (Gibco, cat# 31053) supplemented with 10% heat‐inactivated fetal bovine serum (FBS), 10 mM HEPES, 1 mM sodium pyruvate, 2 mM L‐glutamine, and 100 U/mL penicillin–streptomycin. The day before the experiment MIN6 cells were switched to DMEM supplemented as above but containing 11 mM glucose. The glucose concentration used for upkeeping condition was 11.1 mM for INS1E cells, which corresponds to approximately V½ for glucose‐stimulated insulin secretion [60] and 5.5 mM for αTC1‐9 cells. Although dose–response data are limited for αTC1‐9 cells, previous secretion assays indicate that glucagon release is already inhibited at ~6 mM glucose [47]. When needed, αTC1‐9 cells were transfected with NPY‐EGFP (EGFP‐tagged neuropeptide Y plasmid, a gift from Justin Taraska; Addgene plasmid# 74629 [http://n2t.net/addgene:74629; RRID: Addgene_74629]) [29]. INS‐1E cells were transfected with C‐pep‐EGFP [61] and 1.5 μg of plasmid DNA was used per well for both the transfections. The transfections were performed by plating αTC1‐9 cells on 22‐mm glass‐bottom dishes (WillCo Wells, cat# HBST‐3522) and INS‐1E cells on ibiTreat μ‐Dish 35 mm plates (Ibidi, cat# 81156). The following day, transfection was carried out using Lipofectamine 2000 (Life Technologies) according to the manufacturer's instructions. Cells were imaged 24 h post‐transfection.

### 4.2 Live‐Cell Imaging

For iMSD and SPT experiments, GSGs and ISGs were labeled with ZIGIR, a fluorescent probe that targets zinc‐rich granules [28]. αTC1‐9 and INS‐1E cells were plated on 22‐mm glass‐bottom dishes and ibiTreat μ‐Dish 35 mm plates, respectively, and allowed to adhere overnight before labeling and imaging. For labeling, cells were incubated with 1 μM ZIGIR in upkeeping medium and after 15 min of incubation, unless otherwise specified, imaging was performed. For assessing cytoskeletal components depolymerization, cells were treated for 30 min with 10 μM SiR‐Tubulin (Spirochrome, cat# SC002 [62]) to label microtubules or with 10 μM SiR‐Actin (Spirochrome, cat# SC001 [62]) to label actin filaments. For these treatments, 10 μM Nocodazole (Sigma‐Aldrich, cat# 487928) and 10 μM Latrunculin‐B (Sigma‐Aldrich, cat# 428020) were added to upkeeping medium 30 min before imaging to disrupt microtubules and actin filaments, respectively. To label granules in these experiments, 1 μM ZIGIR was added concomitantly with the cytoskeletal polymerization inhibitors. In both cases, image acquisition was completed within 1 h and 30 min starting from imaging. For the labeling comparison experiments (Figures 2 and S3) between ZIGIR and EGFP‐tagged markers for both αTC1‐9 cells and INS‐1E cells through iMSD, image stacks were acquired using a Leica SP5 inverted confocal microscope. The labeling method comparison was conducted in cells labeled with ZIGIR or transfected with C‐pep‐EGFP (INS‐1E cells) or NPY‐EGFP (αTC1‐9 cells) exciting ZIGIR at 514 nm and EGFP‐tagged probes at 488 nm and collecting the signal between 525 and 700 nm and between 500 and 600 nm, respectively. A 100× oil‐immersion objective (N.A. 1.3) was used, with the pinhole aperture set to 1 Airy. Each stack consisted of 500 frames, acquired with a 204 ms acquisition time per frame and a 1400 Hz line frequency. Other iMSD and SPT experiments were conducted using two additional confocal microscopes with equivalent tracking capabilities. Imaging was performed on an inverted Zeiss LSM 800 confocal microscope (Jena, Germany), exciting ZIGIR at 561 nm and collecting fluorescence between 590 and 700 nm. The pinhole aperture was set to 1 Airy (53 μm), using a 63× (N.A. 1.4) oil objective. Each stack consisted of 500 frames, acquired with a 204 ms acquisition time per frame. Moreover, the Zeiss confocal microscope was used to perform a colocalization study in live cells exciting sequentially ZIGIR at 561 nm and EGFP at 488, collecting the signal between 550 nm and 700 nm and between 400 nm and 550 nm, respectively. Additionally, a Leica Stellaris 8 confocal microscope was used, exciting ZIGIR at 545 nm and collecting fluorescence between 565 and 732 nm. Each stack consisted of 500 frames at 256 × 256 pixels resolution, with an acquisition time of 198 ms per frame. Live‐cell imaging was performed at 37°C under controlled CO_2_ conditions.

### 4.3 Immunostaining and Colocalization Analysis

αTC1‐9 cells were transfected with NPY‐EGFP and plated on glass coverslips. After 24 h, the cells were fixed with 4% paraformaldehyde (PFA) for 30 min at room temperature (RT), followed by permeabilization with PBS containing 0.1% Triton X‐100 (PBST) for 10 min at RT. To block non‐specific binding, cells were incubated with 2% bovine serum albumin (BSA) for 30 min at RT. For immunostaining, cells were incubated with a mouse anti‐glucagon primary antibody (Boster, cat# MA1047) diluted 1:200 for 2 h at 37°C, followed by incubation with an anti‐mouse Alexa Fluor 647 secondary antibody (ThermoFisher, cat# A31571) diluted 1:500 for 1 h at RT in the dark. Colocalization analysis was performed to validate the specificity of glucagon granule labeling. Colocalization comparison was first performed between ZIGIR and NPY‐EGFP in live αTC1‐9 cells, followed by a comparison between NPY‐EGFP and anti‐glucagon in fixed samples. This two‐step approach was necessary because ZIGIR cannot be used on fixed samples [28]. Pearson correlation and Manders coefficients were calculated using the BIOP JACoP plugin for ImageJ, applying the Otsu method for thresholding. For STORM imaging, the same fixation protocol previously described was applied to both αTC1‐9 cells and INS‐1E cells. For STORM immunostaining, αTC1‐9 cells were incubated with anti‐glucagon primary antibody as previously described, while INS‐1E cells were incubated with an anti‐insulin primary antibody (Immunological Sciences, cat# AB‐84377) diluted 1:200 at 4°C overnight. Both primary antibody incubations were followed by incubation with an anti‐mouse Alexa Fluor 647 secondary antibody (ThermoFisher, cat# A31571), diluted 1:500, for 1 h at RT in the dark.

### 4.4 dSTORM Imaging of Insulin and Glucagon Secretory Granules

A Nikon N‐STORM TIRF microscope (Nikon Instruments), equipped with a 100× oil immersion objective (CFI Apo TIRF 100×, NA 1.49, oil; Nikon), was used to acquire 20,000 frames in TIRF illumination mode. Images were captured at a digital resolution of 256 × 256 pixels (crop), with a pixel size of 158.7 nm. Acquisitions consisted of 5000 cycles, each including one activation frame with a 405 nm excitation laser, followed by three readout frames with a 647 nm excitation laser. Excitation intensities, measured at the objective, were ~0.5–1 kW/cm^2^ for the 647 nm readout laser (MPB Communications) and ~35 W/cm^2^ for the 405 nm activation laser (Oxius LBX‐405). Images were detected using an EMCCD camera (Andor iXon DU‐897; Andor Technologies) with EM gain set to 300 and a temporal resolution of 30 ms. The STORM imaging buffer was prepared by filtering and storing at 4°C a solution of 690 μL of 50 mM Tris buffer (pH 8.0), containing 10 mM NaCl and 10% w/v glucose. Immediately before use, 3.5 μL of GLOX solution (described below) and 3.5 μL of 2‐mercaptoethanol were added. The final solution was then applied to the petri dishes, which were sealed with aluminum tape. The GLOX solution was prepared by dissolving 14 mg of glucose oxidase and 50 μL of catalase (17 mg/mL) in 200 μL of STORM imaging buffer. It was stored at 4°C for a maximum of 14 days. Acquired dSTORM stacks were processed using ThunderStorm, a Fiji (ImageJ) plugin for PALM and STORM data analysis. First, acquisition properties were configured in the “Camera setup” menu: pixel size = 158.7 nm, photoelectrons per A/D count = 2.5, base level = 100 counts, and EM gain = 300. Localization was performed using the default algorithm, and results were refined through post‐filtering in ThunderStorm, including (a) removal of the first 500 frames; (b) drift correction by correlation; (c) merging of reactivated molecules (max distance: 20 nm, max off frames: 1, unlimited frames per molecule) (d) removal of localizations with sigma > 130 nm or uncertainty > 40 nm. Filtered molecule localizations were first analyzed using a ClusDoC algorithm for clustering [63] installed in MATLAB, based on the DBSCAN algorithm for clusterization. The parameters for clusterization were set as: epsilon = 50 nm and minPts = 8, respectively representing the maximum distance between two points for them to be considered neighbors and the minimum number of neighboring points required to form a cluster. Each identified cluster was considered a secretory granule. The cluster contour is determined by creating a 2D histogram of localization points within the cluster as described in the ClusDoc reference paper [63]. The boundary of this region is used as the cluster contour for measuring single granule diameter, approximating it as a circle through a custom‐made MATLAB script.

### 4.5 Data Analysis: iMSD

A custom MATLAB (MathWorks Inc., Natick, MA) script was adapted to extract dynamic and structural information from iMSD image stacks. This script, fully described in Ferri et al. [14], calculates a spatiotemporal correlation function and determines its width at each lag time in order to extract the iMSD curve.

The resulting iMSD curve describes the average motion law of granules, and can be characterized by two diffusion parameters:

- Anomalous diffusion coefficient (alpha anomalous), which indicates the type of motion: < 1 corresponds to anomalous sub‐diffusion, ≈1 to Brownian motion, and > 1 to super‐diffusion, likely due to guided drifted motion.
- Average diffusion coefficient (*D*_*m*_): Represents the local (or short‐lag time) free diffusivity of granules, potentially within confined regions.

Additionally, the offset of the iMSD curve provides information about granule size. While the offset does not correspond to the actual physical size of the object, it serves as a squared apparent size metric that can be compared across different experimental conditions.

### 4.6 Data Analysis: Single Particle Tracking

SPT analysis was performed using TrackMate, an open‐source ImageJ plugin that enables particle detection and localization, and trajectory reconstruction [38, 39]. TrackMate identified labeled granules in time‐lapse videos, applying a Laplacian of Gaussians (LoG) filter for particle detection and localization. The LoG detector settings included an estimated object diameter of 0.3 μm, a quality threshold optimized per dataset, median filter preprocessing, and subpixel localization. For trajectory reconstruction, we used the LAP tracker [40], which allows for gap‐closing events, but without considering splitting or merging. The tracking parameters were:

- Maximum frame‐to‐frame linking distance: 0.5 μm
- Maximum gap‐closing distance: 0.5 μm
- Maximum frame gap: 3 frames

Custom MATLAB scripts converted TrackMate results (exported as spot statistics) and performed further data analysis and classification (Table S1 for parameters summary). Only trajectories spanning more than five frames were considered for the analysis.

#### 4.6.1 Trajectories Segmentation and Motion Classification

To segment trajectories and classify motion types, we performed two steps: first, we applied a stop‐and‐go analysis based on a velocity threshold (*v*_*t*_). The velocity of each granule was calculated within a moving 5‐frame window across the 500‐frame image stack. If needed, the trajectories were divided into sub‐trajectories with at least 5 spots having this velocity above and below *v*_*t*_, and the (sub)trajectories (meaning both trajectories and sub‐trajectories) were classified as follows:

- Drifted motion (“isdrifted”/go motion [32, 37, 41]): (sub)trajectories with velocities above *v*_*t*_.
- Mixed/stop trajectories: (sub)trajectories with velocity below *v*_*t*_, often containing segments with different motion types.

Based on empirical tests, a velocity threshold *v*_*t*_ = 0.2 μm/s was used. In the second step, MSS‐TAD (moment scaling spectrum—transient arrest of diffusion) analysis [32] was applied to these mixed (sub)trajectories to distinguish between diffusive and blocked motion. Briefly, as in Durso et al. [37] and Marchetti et al. [32], the mean square displacement (MSD) and different order displacement moments (1st to 6th order) were computed up to a lag time corresponding to the maximum between one‐quarter of the number of trajectory frames and five frames. If the trajectory was considered “mixed” based on the behavior of these functions, the algorithm looked for “confinement zones” by checking the likelihood *L*_*c*_ that the local diffusivity was too low with respect to the average. To identify these confinement zones, we adopted a seed‐and‐threshold strategy [64] (https://svi.nl/SeedAndThreshold) rather than setting hard thresholds for minimum *L*_*c*_ and minimum duration in frames (*t*_*c*_). The segmentation process included:

1. Seed zone selection: Using an initial likelihood threshold *L*_*c*1_ = 1.8 and keeping only segments longer than *t*_*c*1_ = 5 (including first and last frames).
2. Region growing: Extending seed zones using a second, lower likelihood threshold *L*_*c*2_ = 1.4, retaining segments longer than *t*_*c*2_ (7).

The final sub‐trajectory classification was performed according to the MSS‐TAD framework, as previously described in Marchetti et al. [32], excluding the “TAD” category for sub‐trajectories, and the (sub)trajectories were finally summarized into three categories (Table S1 for parameters):

- Blocked: stationary, almost immobile trajectories. The “blocked” category includes the “immobile” (sub)trajectories in Marchetti et al. [32].
- Diffusive: exploratory motion possibly within a confined region. The “diffusive” category includes all the (sub)trajectories classified as “slow” and “fast” in Marchetti et al. [32], which is different with respect to Durso et al. [37]. However, in this work we consider higher limits to discern “immobile” versus “slow” versus “fast” (sub)trajectories for diffusivity: 2 × 10^−3^ and 10^−2^ μm^2^/s here (D_block and D‐lim in Table S1, respectively) vs. 10^−3^ and 2 × 10^−3^ μm^2^/s in Durso et al. [37], respectively.
- Drifted: fast, directional transport‐associated motion. The “drifted” category includes the “drifted” (sub)trajectories deriving from both analysis steps.

Finally, we expressed results as “length” percentages, representing the fraction of the total number of spots within (sub)trajectories classified as blocked, diffusive, or drifted.

Moreover, in some cases we determined the experimental diffusivity—*γ* anomalous parameter joint distribution (see the final part of the section “SPT‐based classification of granule dynamics under upkeeping conditions”) starting from all the (sub)trajectories, like in Marchetti et al. [32].

### 4.7 Stimulation Protocols

Granule motion dynamics have been analyzed under secretion conditions as per protocols contained in literature [14, 47]. These protocols require a KRBH working buffer, based on the Krebs‐Ringer physiological buffer, composed of 140 mM NaCl, 3.6 mM KCl, 0.5 mM NaH_2_PO_4_, 0.5 mM MgSO_4_, 10 mM HEPES, 5 mM NaHCO_3_, 1.5 mM CaCl_2_, 0.1% BSA, pH 7.4.

#### 4.7.1 INS‐1E Stimulation Protocol

INS‐1E cells were seeded onto Ibidi plates and cultured in upkeeping conditions until they reached 75%–80% confluence on the day of the experiment. Cells were washed twice with KRBH supplemented with 2.5 mM glucose and pre‐incubated in this low‐glucose medium with 1 μM ZIGIR for granule labeling for 30 min at 37°C and 5% CO₂. Following pre‐incubation, confocal imaging was performed under these low‐glucose conditions for up to 30 min. To induce secretion, glucose was added to the KRBH medium to reach 16.7 mM (high‐glucose condition), and, after 3 min incubation, imaging continued for an additional 30 min. For a negative control, the same protocol was applied to αTC1‐9 cells, which were plated on WillCo plates instead of Ibidi plates.

#### 4.7.2 αTC1‐9 Stimulation Protocol

αTC1‐9 cells were maintained in growth medium supplemented with glucose to reach a final concentration of 15 mM for 5–7 days prior to the experiments described below, to increase glucagon secretion in hypoglycemic conditions as previously shown [65]. For glucagon secretion experiments [47], cells were seeded on WillCo plates two days before the experiment (Day 0) to reach 75%–80% confluence by the day of the experiment.

- Day 1 (Starvation): Growth medium (DMEM, see Cell Culture section) was replaced by DMEM (Pan Biotech, cat# P04‐01548) supplemented as described above but with 2.5 mM glucose.
- Day 2 (Experiment): Cells were washed twice with KRBH + 2.5 mM glucose and pre‐incubated in this medium at 37°C and 5% CO_2_ for 45–60 min. Image stacks were acquired during this period for the control condition, with 1 μM ZIGIR used for granule labeling 15 min before imaging.

To stimulate glucagon release, after pre‐incubation, cells were washed with KRBH (0 mM glucose), then incubated in KRBH supplemented with 1 mM glucose, along with 1 μM ZIGIR for granule labeling. Image acquisition was completed within 30–40 min to pair the stimulation of insulin granule release. For the negative control, the same protocol was applied to INS1‐E cells. To assess glucagon secretion, αTC1‐9 cells were cultured in 24‐well tissue culture plates and subjected to the above‐described stimulation protocol. A glucose concentration of 1 mM was used to mimic hypoglycemic condition, and 6 mM glucose served as control. Supernatants were collected after 45 min from the stimuli and glucagon content was quantified with a Glucagon ELISA assay (Mercodia, #10‐1281‐01). Glucagon secretion data were normalized on protein content measured by Pierce BCA protein assay kit (ThermoFisher, #23225).

### 4.8 Statistical Analysis

To assess data distribution, we applied the Shapiro–Wilk normality tests. For comparing normally distributed data, an unpaired t‐test was used, while for non‐normally distributed data, we employed the Mann–Whitney nonparametric tests. In cases involving multiple group comparisons, for trajectory categories, we performed multiple t tests using the Holm‐Sidak correction method (alpha = 0.05); each row of data was analyzed individually, without assuming a consistent SD. *p < 0.05, ***p < 0.001, ****p < 0.0001; non‐significant differences are not shown. All tests were performed using GraphPad Prism version 8.0.1 for Windows, GraphPad Software, San Diego, California USA, www.graphpad.com.

## Author Contributions

**S.G**. investigation, formal analysis, visualization, writing – original draft, writing – review and editing. **V.D.L**. investigation, formal analysis, visualization, writing – original draft, writing – review and editing. **G.F**. investigation, formal analysis. **L.A.P**. investigation, formal analysis. **M.T**. investigation, formal analysis. **P.M**. conceptualization, writing – original draft, writing – review and editing. **S.L**. conceptualization, software development, formal analysis, writing – original draft, writing – review and editing. **F.C**. conceptualization, funding acquisition, supervision, writing – original draft, writing – review and editing.

## Ethics Statement

The authors have nothing to report.

## Conflicts of Interest

The authors declare no conflicts of interest.

## Peer Review

The peer review history for this article is available at https://www.webofscience.com/api/gateway/wos/peer‐review/10.1111/tra.70019.

## Supporting information


**Data S1:** tra70019‐sup‐0001‐supinfo.docx.

## Data Availability

The authors confirm that the data supporting the findings of this study are available within the article and its Supporting Information.

## References

[1] S. M. Hartig and A. R. Cox , “Paracrine Signaling in Islet Function and Survival,” Journal of Molecular Medicine 98, no. 4 (2020): 451–467, 10.1007/s00109-020-01887-x. 32067063 PMC7899133

[2] A. Wendt and L. Eliasson , “Pancreatic Alpha Cells and Glucagon Secretion: Novel Functions and Targets in Glucose Homeostasis,” Current Opinion in Pharmacology 63 (2022): 102199, 10.1016/j.coph.2022.102199. 35245797

[3] S. L. Aronoff , K. Berkowitz , B. Shreiner , and L. Want , “Glucose Metabolism and Regulation: Beyond Insulin and Glucagon,” Diabetes Spectrum: A Publication of the American Diabetes Association 17, no. 3 (2004): 183–190, 10.2337/diaspect.17.3.183.

[4] M. F. Brereton , E. Vergari , Q. Zhang , and A. Clark , “Alpha‐, Delta‐ and PP‐Cells: Are They the Architectural Cornerstones of Islet Structure and Co‐Ordination?,” Journal of Histochemistry and Cytochemistry 63, no. 8 (2015): 575–591, 10.1369/0022155415583535. 26216135 PMC4530398

[5] M. Omar‐Hmeadi and O. Idevall‐Hagren , “Insulin Granule Biogenesis and Exocytosis,” Cellular and Molecular Life Sciences 78, no. 5 (2021): 1957–1970, 10.1007/s00018-020-03688-4. 33146746 PMC7966131

[6] S. Barg , “Mechanisms of Exocytosis in Insulin‐Secreting B‐Cells and Glucagon‐Secreting A‐Cells,” Pharmacology & Toxicology 92, no. 1 (2003): 3–13, 10.1034/j.1600-0773.2003.920102.x. 12710591

[7] E. D. Abel , A. L. Gloyn , C. Evans‐Molina , et al., “Diabetes Mellitus—Progress and Opportunities in the Evolving Epidemic,” Cell 187, no. 15 (2024): 3789–3820, 10.1016/J.CELL.2024.06.029. 39059357 PMC11299851

[8] P. Rorsman and E. Renström , “Insulin Granule Dynamics in Pancreatic Beta Cells,” Diabetologia 46, no. 8 (2003): 1029–1045, 10.1007/s00125-003-1153-1. 12879249

[9] P. Hoboth , A. Müller , A. Ivanova , et al., “Aged Insulin Granules Display Reduced Microtubule‐Dependent Mobility and Are Disposed Within Actin‐Positive Multigranular Bodies,” Proceedings of the National Academy of Sciences of the United States of America 112, no. 7 (2015): E667–E676, 10.1073/pnas.1409542112. 25646459 PMC4343180

[10] E. Fava , J. Dehghany , J. Ouwendijk , et al., “Novel Standards in the Measurement of Rat Insulin Granules Combining Electron Microscopy, High‐Content Image Analysis and in Silico Modelling,” Diabetologia 55, no. 4 (2012): 1013–1023, 10.1007/s00125-011-2438-4. 22252472 PMC3296007

[11] X. Zhu , R. Hu , M. Brissova , et al., “Microtubules Negatively Regulate Insulin Secretion in Pancreatic β Cells,” Developmental Cell 34, no. 6 (2015): 656–668, 10.1016/j.devcel.2015.08.020. 26418295 PMC4594944

[12] M. Ohara‐Imaizumi , C. Nishiwaki , T. Kikuta , S. Nagai , Y. Nakamichi , and S. Nagamatsu , “TIRF Imaging of Docking and Fusion of Single Insulin Granule Motion in Primary Rat Pancreatic β‐Cells: Different Behaviour of Granule Motion Between Normal and Goto‐Kakizaki Diabetic Rat β‐Cells,” Biochemical Journal 381, no. 1 (2004): 13–18, 10.1042/BJ20040434. 15128287 PMC1133756

[13] L. Ma , V. P. Bindokast , A. Kuznetsov , et al., “Direct Imaging Shows That Insulin Granule Exocytosis Occurs by Complete Vesicle Fusion,” Proceedings of the National Academy of Sciences of the United States of America 101, no. 25 (2004): 9266–9271, 10.1073/pnas.0403201101. 15197259 PMC438965

[14] G. Ferri , L. Digiacomo , Z. Lavagnino , et al., “Insulin Secretory Granules Labelled With Phogrin‐Fluorescent Proteins Show Alterations in Size, Mobility and Responsiveness to Glucose Stimulation in Living β‐Cells,” Scientific Reports 9, no. 1 (2019): 2890, 10.1038/s41598-019-39329-5. 30814595 PMC6393586

[15] A. T. Heaslip , S. R. Nelson , A. T. Lombardo , S. B. Previs , J. Armstrong , and D. M. Warshaw , “Cytoskeletal Dependence of Insulin Granule Movement Dynamics in INS‐1 Beta‐Cells in Response to Glucose,” PLoS One 9, no. 10 (2014): e109082, 10.1371/journal.pone.0109082. 25310693 PMC4195697

[16] S. M. A. Tabei , S. Burov , H. Y. Kim , et al., “Intracellular Transport of Insulin Granules Is a Subordinated Random Walk,” Proceedings of the National Academy of Sciences of the United States of America 110, no. 13 (2013): 4911–4916, 10.1073/pnas.1221962110. 23479621 PMC3612641

[17] M. Hao , X. Li , M. A. Rizzo , J. V. Rocheleau , B. M. Dawant , and D. W. Piston , “Regulation of Two Insulin Granule Populations Within the Reserve Pool by Distinct Calcium Sources,” Journal of Cell Science 118, no. 24 (2005): 5873–5884, 10.1242/jcs.02684. 16317050

[18] R. Ivarsson , S. Obermüller , G. A. Rutter , J. Galvanovskis , and E. Renström , “Temperature‐Sensitive Random Insulin Granule Diffusion Is a Prerequisite for Recruiting Granules for Release,” Traffic 5, no. 10 (2004): 750–762, 10.1111/j.1600-0854.2004.00216.x. 15355511

[19] T. K. Bratanova‐Tochkova , H. Cheng , S. Daniel , et al., “Triggering and Augmentation Mechanisms, Granule Pools, and Biphasic Insulin Secretion,” Diabetes 51 (2002): S83–S90, 10.2337/diabetes.51.2007.s83. 11815463

[20] S. Daniel , M. Noda , S. G. Straub , and G. W. G. Sharp , “Identification of the Docked Granule Pool Responsible for the First Phase of Glucose‐Stimulated Insulin Secretion,” Diabetes 48, no. 9 (1999): 1686–1690, 10.2337/diabetes.48.9.1686. 10480595

[21] W. Li , A. Li , B. Yu , et al., “In Situ Structure of Actin Remodeling During Glucose‐Stimulated Insulin Secretion Using Cryo‐Electron Tomography,” Nature Communications 15, no. 1 (2024): 1311, 10.1038/s41467-024-45648-7. PMC10861521 38346988

[22] M. A. Kalwat and D. C. Thurmond , “Signaling Mechanisms of Glucose‐Induced F‐Actin Remodeling in Pancreatic Islet β Cells,” Experimental and Molecular Medicine 45, no. 8 (2013): e37, 10.1038/emm.2013.73. 23969997 PMC3789261

[23] A. Quinault , B. Gausseres , D. Bailbe , et al., “Disrupted Dynamics of F‐Actin and Insulin Granule Fusion in INS‐1 832/13 Beta‐Cells Exposed to Glucotoxicity: Partial Restoration by Glucagon‐Like Peptide 1,” Biochimica et Biophysica Acta ‐ Molecular Basis of Disease 1862, no. 8 (2016): 1401–1411, 10.1016/j.bbadis.2016.04.007. 27101990

[24] D. C. Thurmond , C. Gonelle‐Gispert , M. Furukawa , P. A. Halban , and J. E. Pessin , “Glucose‐Stimulated Insulin Secretion Is Coupled to the Interaction of Actin With the t‐SNARE (Target Membrane Soluble N‐Ethylmaleimide‐Sensitive Factor Attachment Protein Receptor Protein) Complex,” Molecular Endocrinology 17, no. 4 (2003): 732–742, 10.1210/me.2002-0333. 12554769

[25] X. W. Ng , Y. H. Chung , F. Asadi , C. Kong , A. Ustione , and D. W. Piston , “RhoA as a Signaling Hub Controlling Glucagon Secretion From Pancreatic a‐Cells,” Diabetes 71, no. 11 (2022): 2384–2394, 10.2337/db21-1010. 35904939 PMC9630081

[26] X. Liu , T. dos Santos , A. F. Spigelman , et al., “TMEM55A‐Mediated PI5P Signaling Regulates α‐Cell Actin Depolymerization and Glucagon Secretion,” Preprint posted online December 17, 2024, 10.1101/2024.12.16.628242. PMC12176968 40140059

[27] S. Yokawa , T. Furuno , T. Suzuki , Y. Inoh , R. Suzuki , and N. Hirashima , “Effect of Cell Adhesion Molecule 1 Expression on Intracellular Granule Movement in Pancreatic α Cells,” Cell Biochemistry and Biophysics 74, no. 3 (2016): 391–398, 10.1007/s12013-016-0737-6. 27262873

[28] E. H. Ghazvini Zadeh , Z. J. Huang , J. Xia , D. Li , H. W. Davidson , and W. h. Li , “ZIGIR, a Granule‐Specific Zn2+ Indicator, Reveals Human Islet α Cell Heterogeneity,” Cell Reports 32, no. 2 (2020): 107904, 10.1016/j.celrep.2020.107904. 32668245 PMC7410119

[29] M. Omar‐Hmeadi , P. E. Lund , N. R. Gandasi , A. Tengholm , and S. Barg , “Paracrine Control of α‐Cell Glucagon Exocytosis Is Compromised in Human Type‐2 Diabetes,” Nature Communications 11, no. 1 (2020): 1896, 10.1038/s41467-020-15717-8. PMC7171169 32312960

[30] C. Di Rienzo , E. Gratton , F. Beltram , and F. Cardarelli , “Fast Spatiotemporal Correlation Spectroscopy to Determine Protein Lateral Diffusion Laws in Live Cell Membranes,” Proceedings of the National Academy of Sciences of the United States of America 110, no. 30 (2013): 12307–12312, 10.1073/pnas.1222097110. 23836651 PMC3725058

[31] L. Digiacomo , M. A. Digman , E. Gratton , and G. Caracciolo , “Development of an Image Mean Square Displacement (iMSD)‐Based Method as a Novel Approach to Study the Intracellular Trafficking of Nanoparticles,” Acta Biomaterialia 42 (2016): 189–198, 10.1016/j.actbio.2016.07.031. 27449340 PMC5483853

[32] L. Marchetti , A. Callegari , S. Luin , et al., “Ligand Signature in the Membrane Dynamics of Single TrkA Receptor Molecules,” Journal of Cell Science 126, no. 19 (2013): 4445–4456, 10.1242/jcs.129916. 23886941

[33] D. Nam , J. Mantell , D. Bull , P. Verkade , and A. Achim , “A Novel Framework for Segmentation of Secretory Granules in Electron Micrographs,” Medical Image Analysis 18, no. 2 (2014): 411–424, 10.1016/j.media.2013.12.008. 24444668

[34] S. Asai , J. Moravcová , L. Žáková , et al., “Characterization of Insulin Crystalline Form in Isolated β‐Cell Secretory Granules,” Open Biology 12, no. 12 (2022): 220322, 10.1098/rsob.220322. 36541100 PMC9768635

[35] G. Ferri , F. Azzarello , F. D'autilia , and F. Cardarelli , “Probing Structural and Dynamic Properties of Trafficking Subcellular Nanostructures by Spatiotemporal Fluctuation Spectroscopy,” Journal of Visualized Experiments 2021, no. 174 (2021): e62790, 10.3791/62790. 34459819

[36] L. Digiacomo , F. D'Autilia , W. Durso , P. M. Tentori , G. Caracciolo , and F. Cardarelli , “Dynamic Fingerprinting of Sub‐Cellular Nanostructures by Image Mean Square Displacement Analysis,” Scientific Reports 7, no. 1 (2017): 14836, 10.1038/s41598-017-13865-4. 29093485 PMC5665924

[37] W. Durso , M. Martins , L. Marchetti , F. Cremisi , S. Luin , and F. Cardarelli , “Lysosome Dynamic Properties During Neuronal Stem Cell Differentiation Studied by Spatiotemporal Fluctuation Spectroscopy and Organelle Tracking,” International Journal of Molecular Sciences 21, no. 9 (2020): 3397, 10.3390/ijms21093397. 32403391 PMC7247004

[38] J. Y. Tinevez , N. Perry , J. Schindelin , et al., “TrackMate: An Open and Extensible Platform for Single‐Particle Tracking,” Methods 115 (2017): 80–90, 10.1016/j.ymeth.2016.09.016. 27713081

[39] D. Ershov , M. S. Phan , J. W. Pylvänäinen , et al., “TrackMate 7: Integrating State‐Of‐The‐Art Segmentation Algorithms Into Tracking Pipelines,” Nature Methods 19, no. 7 (2022): 829–832, 10.1038/s41592-022-01507-1. 35654950

[40] K. Jaqaman , D. Loerke , M. Mettlen , et al., “Robust Single‐Particle Tracking in Live‐Cell Time‐Lapse Sequences,” Nature Methods 5, no. 8 (2008): 695–702, 10.1038/nmeth.1237. 18641657 PMC2747604

[41] T. De Nadai , L. Marchetti , C. Di Rienzo , et al., “Precursor and Mature NGF Live Tracking: One Versus Many at a Time in the Axons,” Scientific Reports 6 (2016): 6, 10.1038/srep20272. 26829890 PMC4735336

[42] D. Convertino , F. Fabbri , N. Mishra , et al., “Graphene Promotes Axon Elongation Through Local Stall of Nerve Growth Factor Signaling Endosomes,” Nano Letters 20, no. 5 (2020): 3633–3641, 10.1021/acs.nanolett.0c00571. 32208704

[43] A. Falconieri , S. De Vincentiis , V. Cappello , et al., “Axonal Plasticity in Response to Active Forces Generated Through Magnetic Nano‐Pulling,” Cell Reports 42, no. 1 (2023): 111912, 10.1016/j.celrep.2022.111912. 36640304 PMC9902337

[44] K. Nakashima , Y. Kanda , Y. Hirokawa , F. Kawasaki , M. Matsuki , and K. Kaku , “MIN6 Is Not a Pure Beta Cell Line but a Mixed Cell Line With Other Pancreatic Endocrine Hormones,” Endocrine Journal 56, no. 1 (2009): 45–53, 10.1507/endocrj.K08E-172. 18845907

[45] N. I. Mourad , M. Nenquin , and J. C. Henquin , “Amplification of Insulin Secretion by Acetylcholine or Phorbol Ester Is Independent of β‐Cell Microfilaments and Distinct From Metabolic Amplification,” Molecular and Cellular Endocrinology 367, no. 1‐2 (2013): 11–20, 10.1016/j.mce.2012.12.002. 23246352

[46] A. Ivanova , Y. Kalaidzidis , R. Dirkx , et al., “Age‐Dependent Labeling and Imaging of Insulin Secretory Granules,” Diabetes 62, no. 11 (2013): 3687–3696, 10.2337/db12-1819. 23929935 PMC3806613

[47] A. Veprik , G. Denwood , D. Liu , et al., “Acetyl‐CoA‐Carboxylase 1 (ACC1) Plays a Critical Role in Glucagon Secretion,” Communications Biology 5, no. 1 (2022): 238, 10.1038/s42003-022-03170-w. 35304577 PMC8933412

[48] A. Müller , D. Schmidt , C. S. Xu , et al., “3D FIB‐SEM Reconstruction of Microtubule‐Organelle Interaction in Whole Primary Mouse β Cells,” Journal of Cell Biology 220, no. 2 (2021): e202010039, 10.1083/JCB.202010039. 33326005 PMC7748794

[49] C. R. Pfeifer , A. Shomorony , M. A. Aronova , et al., “Quantitative Analysis of Mouse Pancreatic Islet Architecture by Serial Block‐Face SEM,” Journal of Structural Biology 189, no. 1 (2015): 44–52, 10.1016/j.jsb.2014.10.013. 25448885 PMC4305430

[50] A. Varadi , T. Tsuboi , L. I. Johnson‐Cadwell , V. J. Allan , and G. A. Rutter , “Kinesin I and Cytoplasmic Dynein Orchestrate Glucose‐Stimulated Insulin‐Containing Vesicle Movements in Clonal MIN6 β‐Cells,” Biochemical and Biophysical Research Communications 311, no. 2 (2003): 272–282, 10.1016/j.bbrc.2003.09.208. 14592410

[51] P. Dzianová , S. Asai , M. Chrudinová , et al., “The Efficiency of Insulin Production and Its Content in Insulin‐Expressing Model β‐Cells Correlate With Their Zn^2+^ Levels: Zn^2+^ Levels in β‐Cells,” Open Biology 10, no. 10 (2020): 200137, 10.1098/rsob.200137. 33081637 PMC7653362

[52] N. Lawlor , A. Youn , R. Kursawe , D. Ucar , and M. L. Stitzel , “Alpha TC1 and Beta‐TC‐6 Genomic Profiling Uncovers Both Shared and Distinct Transcriptional Regulatory Features With Their Primary Islet Counterparts,” Scientific Reports 7, no. 1 (2017): 11959, 10.1038/s41598-017-12335-1. 28931935 PMC5607285

[53] J. Camunas‐Soler , X. Q. Dai , Y. Hang , et al., “Patch‐Seq Links Single‐Cell Transcriptomes to Human Islet Dysfunction in Diabetes,” Cell Metabolism 31, no. 5 (2020): 1017–1031.e4, 10.1016/j.cmet.2020.04.005. 32302527 PMC7398125

[54] X. Q. Dai , J. Camunas‐Soler , L. J. B. Briant , et al., “Heterogenous Impairment of α Cell Function in Type 2 Diabetes Is Linked to Cell Maturation State,” Cell Metabolism 34, no. 2 (2022): 256–268.e5, 10.1016/j.cmet.2021.12.021. 35108513 PMC8852281

[55] R. K. P. Benninger and V. Kravets , “The Physiological Role of β‐Cell Heterogeneity in Pancreatic Islet Function,” Nature Reviews. Endocrinology 18, no. 1 (2022): 9–22, 10.1038/s41574-021-00568-0. PMC8915749 34667280

[56] J. J. Park and Y. P. Loh , “How Peptide Hormone Vesicles Are Transported to the Secretion Site for Exocytosis,” Molecular Endocrinology 22, no. 12 (2008): 2583–2595, 10.1210/me.2008-0209. 18669645 PMC2626200

[57] K. Mizuno , J. S. Ramalho , and T. Izumi , “Exophilin8 Transiently Clusters Insulin Granules at the Actin‐Rich Cell Cortex Prior to Exocytosis,” Molecular Biology of the Cell 22, no. 10 (2011): 1716–1726, 10.1091/mbc.E10-05-0404. 21441305 PMC3093323

[58] C. Desnos , S. Huet , I. Fanget , et al., “Myosin Va Mediates Docking of Secretory Granules at the Plasma Membrane,” Journal of Neuroscience 27, no. 39 (2007): 10636–10645, 10.1523/JNEUROSCI.1228-07.2007. 17898234 PMC6673143

[59] N. R. Gandasi , P. Yin , M. Omar‐Hmeadi , E. Ottosson Laakso , P. Vikman , and S. Barg , “Glucose‐Dependent Granule Docking Limits Insulin Secretion and Is Decreased in Human Type 2 Diabetes,” Cell Metabolism 27, no. 2 (2018): 470–478.e4, 10.1016/j.cmet.2017.12.017. 29414688

[60] A. Merglen , S. Theander , B. Rubi , G. Chaffard , C. B. Wollheim , and P. Maechler , “Glucose Sensitivity and Metabolism‐Secretion Coupling Studied During Two‐Year Continuous Culture in INS‐1E Insulinoma Cells,” Endocrinology 145, no. 2 (2004): 667–678, 10.1210/en.2003-1099. 14592952

[61] M. A. Rizzo , M. A. Magnuson , P. F. Drain , and D. W. Piston , “A Functional Link Between Glucokinase Binding to Insulin Granules and Conformational Alterations in Response to Glucose and Insulin,” Journal of Biological Chemistry 277, no. 37 (2002): 34168–34175, 10.1074/jbc.M112478200. 12101177

[62] G. Lukinavičius , L. Reymond , E. D'Este , et al., “Fluorogenic Probes for Live‐Cell Imaging of the Cytoskeleton,” Nature Methods 11, no. 7 (2014): 731–733, 10.1038/nmeth.2972. 24859753

[63] S. V. Pageon , P. R. Nicovich , M. Mollazade , T. Tabarin , and K. Gaus , “Clus‐DoC: A Combined Cluster Detection and Colocalization Analysis for Single‐Molecule Localization Microscopy Data,” Molecular Biology of the Cell 27, no. 22 (2016): 3627–3636, 10.1091/mbc.E16-07-0478. 27582387 PMC5221594

[64] L. Marchetti , F. Bonsignore , F. Gobbo , et al., “Fast‐Diffusing p75NTR Monomers Support Apoptosis and Growth Cone Collapse by Neurotrophin Ligands,” Proceedings of the National Academy of Sciences of the United States of America 116, no. 43 (2019): 21563–21572, 10.1073/pnas.1902790116. 31515449 PMC6815156

[65] R. McGirr , C. E. Ejbick , D. E. Carter , et al., “Glucose Dependence of the Regulated Secretory Pathway in αTC1‐6 Cells,” Endocrinology 146, no. 10 (2005): 4514–4523, 10.1210/en.2005-0402. 15994347

