# Supplementary material for: Spatiotemporal Analysis of Glucagon Secretory Granule Dynamics
Source: Traffic. 2025 Sep 24;26(7-9):e70019. doi: 10.1111/tra.70019 (PMC12457985; doi:10.1111/tra.70019)
Supplement: Supplementary file 1 — Data S1: tra70019‐sup‐0001‐supinfo.docx. [file TRA-26-e70019-s001.docx]

**Supplementary figures**

**
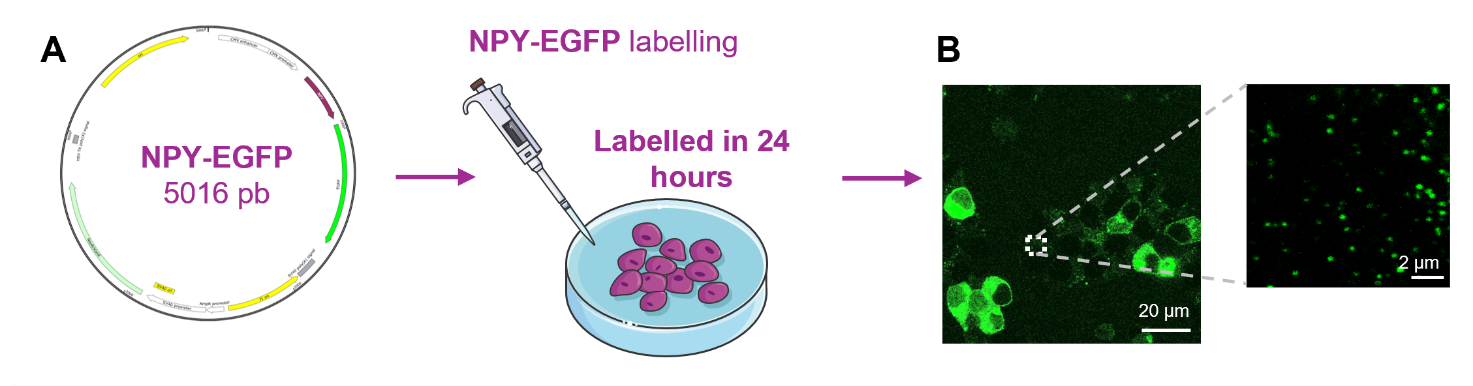
**

**Fig. S1: Glucagon granules labelling method via transfection. (A)** NPY-EGFP transfection labeling protocol for GSGs in αTC1-9 cells, with treatment 24 hours before confocal acquisition. The same protocol was used for C-pep-EGFP labeling for ISGs in INS-1E cells (not shown in the diagram). **(B)** Confocal imaging of NPY-EGFP in αTC1-9 cells shows only a few cells labeled with an acceptable expression level (28.3% ± 8.9%). The diffuse signal observed inside some cells is due to non-specific labeling resulting from NPY-EGFP overexpression. Servier Medical Art (<https://smart.servier.com/>) was used to create part of this figure.


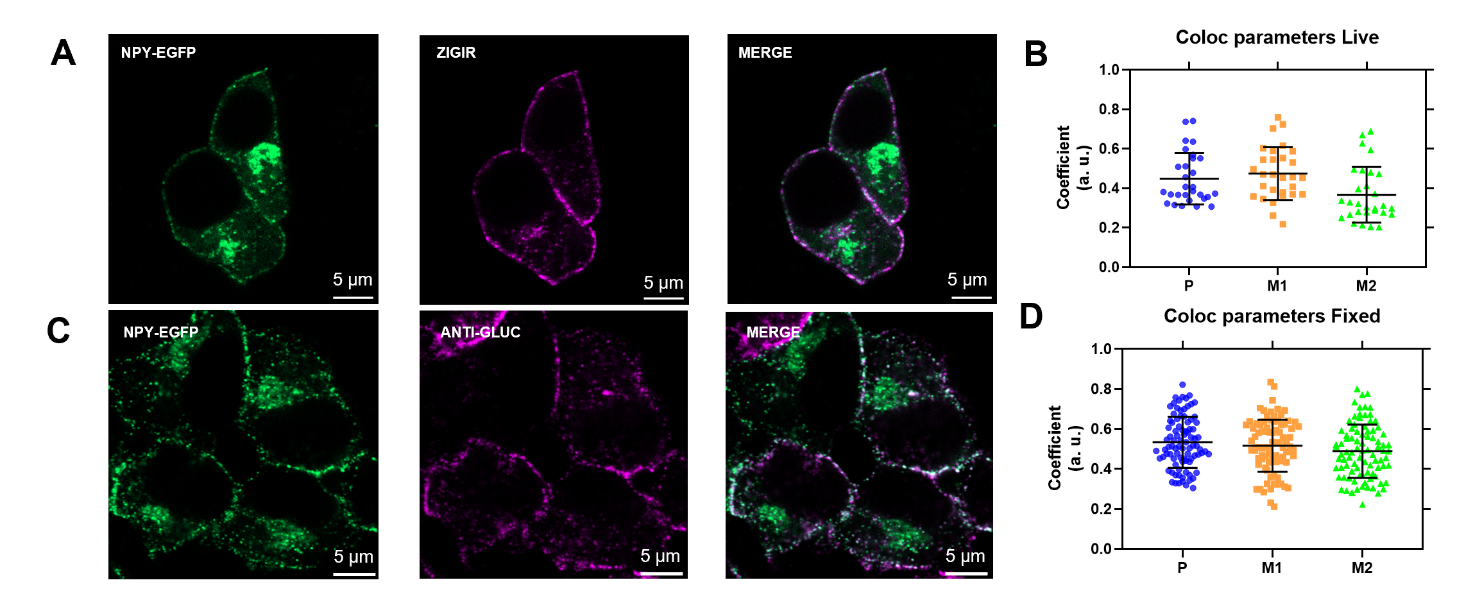


**Fig. S2: Colocalization study in αTC1-9 cells. (A)** Example of colocalization between ZIGIR and NPY-EGFP in living cells, showing differences in labeling due to the lack of specificity of NPY-EGFP, which also labels the Golgi apparatus, as better discernible in the merged image. **(B)** Colocalization parameters for the experiment in panel A, calculated using the BIOP JACOP plugin for different regions of interest (ROI) which can include more than one cell. Dots: in blue, the values of the Pearson's coefficient (P, average 0.42); in orange and green, the Manders colocalization coefficients M1 and M2 (characterizing the fraction of ZIGIR in NPY-EGFP and vice versa, respectively). Scatter plots show Mean ± SD. **(C)** Colocalization between NPY-EGFP and anti-glucagon immunostaining, demonstrating the specificity of the labeling. **(D)** Colocalization parameters for the experiment in panel C on fixed cells; colors and symbols as in panel B, but with M1 and M2 characterizing the fraction of NPY-EGFP into anti-glucagon antibody signal and vice versa, respectively, and average Pearson's coefficient P of 0.53. Scatter plots show Mean ± SD.

**
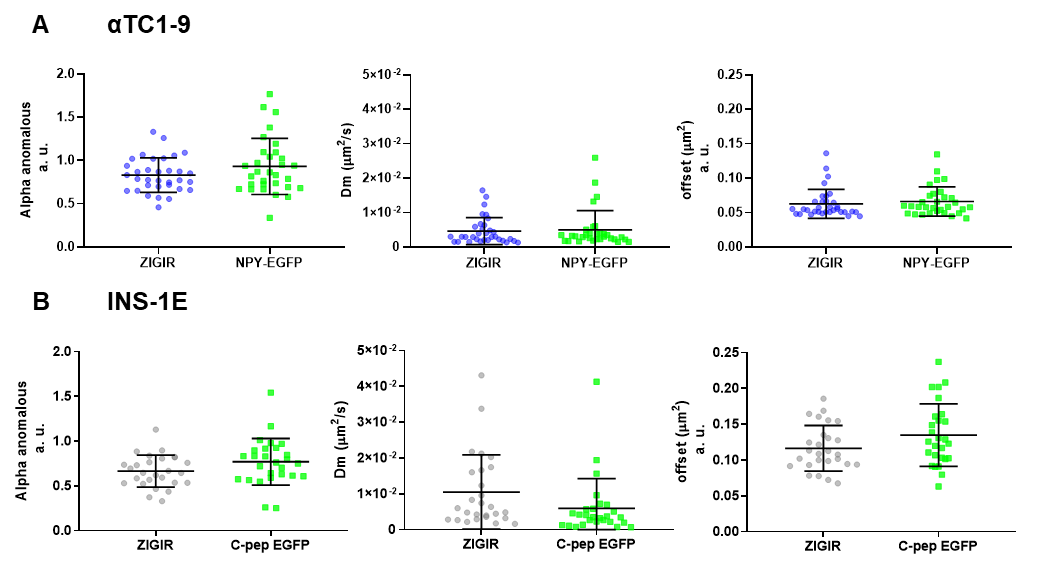
Fig. S3: iMSD analysis with different labelling methods. (A)** Comparison between estimated dynamics parameters using different labelling methods (ZIGIR vs NPY-EGFP) in αTC1-9 cells. In both cases the motion where sub-diffusive even if with an Alpha anomalous coefficient close to 1 (characterizing Brownian motion). There are no significant differences for any dynamic parameter, despite some outliers in both cases. **(B**) Same analysis for different labelling methods (ZIGIR vs C-pep EGFP) in INS-1E cells. No significant differences were present for any parameter. We performed a Shapiro-wilk test to evaluate the normality, followed by a t-test for normally distributed data and Kolmogorov-Smirnov test for not normally distributed data. The absence of differences for the offset parameter indicates that the two labelling techniques produce comparable granules apparent size. Scatter plots with Mean ± SD with each dot shown represent the outcome from a single acquisition.


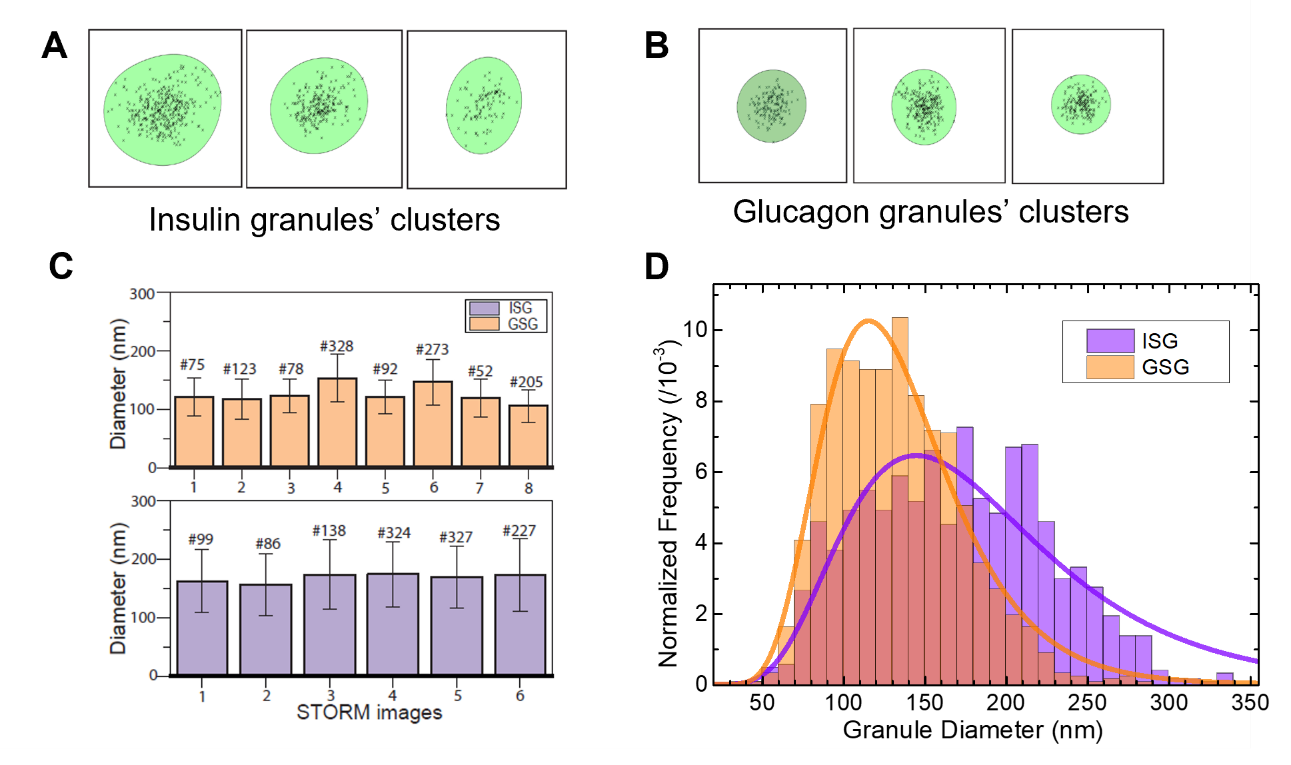


**Fig. S4: STORM imaging of ISGs and GSGs. (A)** Representative ISGs localizations cluster analysis. Green shades represent cluster contours, identified as ISGs composed of localized molecules (black dots) through ClusDoC clustering algorithm. Each square represents a 300x300 nm region of a STORM images. **(B)** Representative GSGs localizations clusters analysis. Green shades represent cluster contours, identified as GSGs composed of localized molecules (black dots) through ClusDoC clustering algorithm. **(C)** Glucagon (orange) and insulin (violet) secretory granule diameters expressed as mean ± SD calculated for each acquired STORM image. The values on top of the bars represent the number of granules identified as cluster of labelled molecules. **(D)** Bars: histogram of secretory granule diameters for ISGs (violet) and GSGs (orange). Transparency was applied to overlapping distributions to maintain visual clarity. Histograms are normalized for area = 1. Lines of corresponding colors are LogNormal fits.


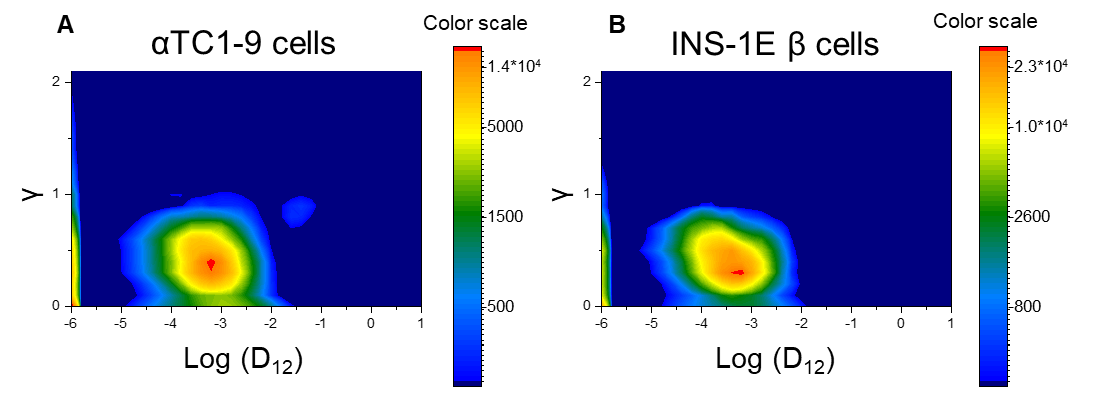


**Fig. S5: Bidimensional distribution of log10(D_12_) on x-axis and γ Anomalous parameter on y-axis for (sub)trajectories obtained by MSS-TAD analysis:** for each (sub)trajectory, D_12_ is the short-lag-time diffusivity and γ is the γ anomalous diffusion coefficient calculated by the MSS analysis, which has a meaning similar to half the Alpha anomalous coefficient. The γ parameter describes the long-term trajectory motion types: γ ≈ 0.5 corresponds to Brownian motion, γ < 0.5 to sub-diffusive motion, and γ > 0.5 to super-diffusive motion. **(A)** Resulting graph for αTC1-9 cells in upkeeping condition, in which the peak is nearby γ ≤ 0.5, indicating a sub-diffusive motion very close to the Brownian one (reflecting the results obtained in the iMSD analysis), and D_12_ ≈ 6.6 * 10^-4^ µm^2^/s. **(B)** Resulting graph for INS-1E cells in upkeeping condition, in which the red peak is well under γ = 0.5, indicating a more sub-diffusive motion and reflecting the result obtained in the iMSD analysis, and D_12_ ≈ 6.6 * 10^-4^ µm^2^/s. The fact that the calculated D_12_ are lower than the corresponding D_m_ calculated by iMSD can reflect either the fact that the peak here is on a logarithmic scale, and the average of the logarithms are usually smaller than the logarithm of the average (even if in this case we are not really considering an average), or the smaller impact of the slower particles on the iMSD results, already cited in the main text. The equality between the D_12_ values for α and β cells reflects the non-significant difference among the D_m_ population highlighted in Fig. 2C.


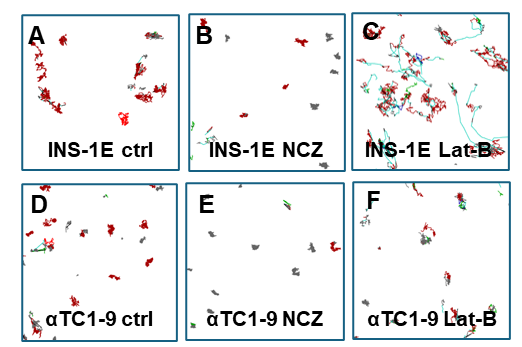


**Fig. S6: SPT classification changes for ISGs and GSGs after microtubules disruption made by Nocodazole (NCZ) and actin filaments disruption made by Latrunculin-B (Lat-B).** Examples of granule trajectories and their classification from the custom-made MATLAB script for β (A-C) and α (D-F) cell models (INS-1E and αTC1-9, respectively) in different conditions: **(A)** IGSs in upkeeping condition (INS-1E ctrl), highlighting many diffusive trajectories. **(B)** NCZ treatment on INS-1E cells (INS-1E NCZ), in which the blocked fraction of trajectories increased reducing both diffusive fraction and drifted motion. **(C)** ISGs under Lat-B treatment (INS-1E Lat-B), in which the diffusive and drifted fraction of the trajectories increased reducing the blocked ones. **(D)** GSGs in upkeeping condition (αTC1-9 ctrl), highlighting many diffusive trajectories **(E)** GSGs upon NCZ treatment (αTC1-9 NCZ), in which the blocked fraction of the trajectories increased reducing both diffusive fraction and drifted motion. **(F)** GSGs upon Lat-B treatment (αTC1-9 Lat-B), in which the blocked fraction of the trajectories increased reducing diffusive fraction but without substantial changes in drifted motion. Gray trajectories are blocked, red trajectories are diffusive, and both cyan and green are drifted (with velocity always above v_t_=0.2 µm/s and classified drifted by the MSS-TAD analysis, respectively).


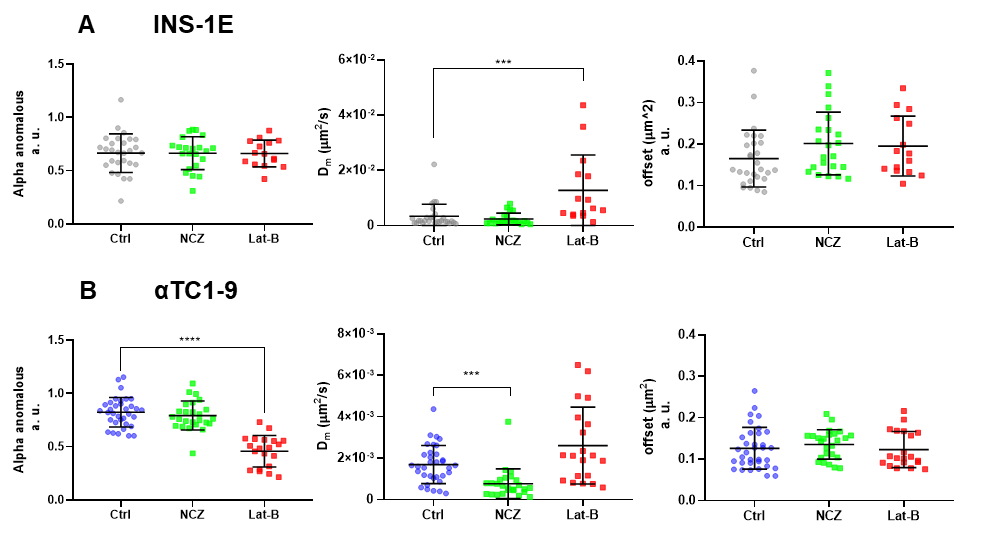


**Fig. S7: iMSD of INS-1E cells and αTC1-9 cells comparing different treatment against cytoskeletal structures. (A)** Scatter plots of the results of iMSD analysis on ISGs, comparing control condition (INs-1E Ctrl, n = 28) with respect to NCZ treatment (INS-1E NCZ, n = 22) for microtubules disruption and Lat-B treatment (INS-1E Lat-B, n = 15) for actin filament disruption. All three conditions produced a mostly sub-diffusive motion, as we can see in the Alpha anomalous parameter graph. For the diffusion coefficient, we observed an increasing in diffusion for the Lat-B treatment, but no difference were pointed out in the NCZ treatment. This is coherent with the SPT results were the diffusive motion increased with respect to the control with Lat-B treatment. As expected, no differences were present in the offset of the ISG in the three conditions, because the drugs are not supposed to alter the size of the granules. **(B)** Scatter plots of the results of iMSD analysis on GSGs, comparing control condition (αTC1-9 Ctrl, n = 34) with respect to NCZ treatment (αTC1-9 NCZ, n = 25) for microtubules disruption and Lat-B treatment (αTC1-9 Lat-B, n = 20) for actin filament disruption. αTC1-9 cells Ctrl condition and NCZ were in a sub-diffusive motion even if quite close to a Brownian one (Alpha anomalous coefficient close to 1), but the comparison with Lat-B condition highlighted a statistical difference amongst the Alpha anomalous coefficient showing a more predominantly sub-diffusive motion in this last case. As we can see in the diffusion coefficient, a sub-diffusive motion does not mean a reduction in the diffusion coefficient (Lat-B). Instead, in the case of NCZ the diffusion coefficient D_m_ is reduced. As expected, no differences were present in the offset of the glucagon granules in the three conditions, because the drugs are not supposed to alter the size. We performed Shapiro-wilk test to evaluate the normality then One-way ANOVA multiple comparison for the statistical differences.


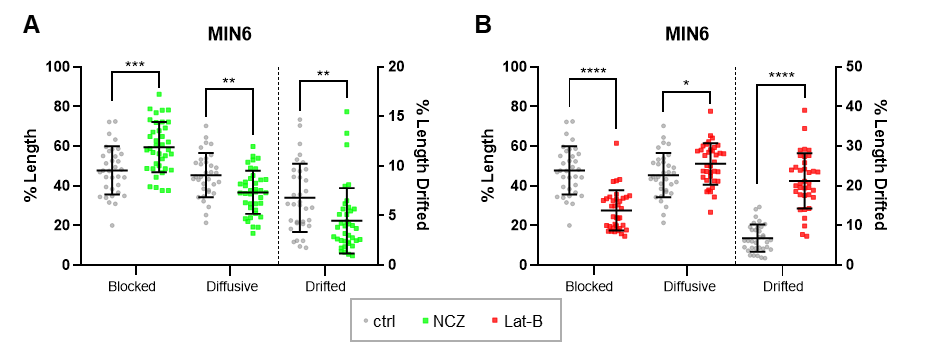


**Fig. S8 SPT classification for ISG after Nocodazole and Latrunculin-B treatment in MIN6 as β cell model. (A)** Scatter plots of the three categories of trajectories about ISGs of MIN6 cells in which we compared the benchmark condition (Ctrl, n = 34) with respect to microtubules alteration (NCZ, n = 38). NCZ treatment imposed an increase in blocked trajectories to the detriment of diffusive and drifted ones. The result denotes a reduction of general motility. Drifted y axis on the right (% Length Drifted). **(B)** Scatter plots of the three categories of trajectories about ISGs of MIN6 cells in which we compared the benchmark condition (Ctrl, n = 34) with respect to actin filaments disruption (Lat-B, n = 36). Lat-B induced a net decrease in blocked trajectories and raised up both diffusive and drifted trajectories. Drifted y axis on the right (% Length Drifted). Results obtained in MIN6 cellular line as β cells model can be coupled with the results obtained in INS-1E cell line, confirming a behaviour related to the ISGs instead of the cellular line itself. We checked normality and we performed multiple t test for the normally distributed data using Holm-Sidak correction method (alpha = 0.05); each row of data was analyzed individually, without assuming a consistent SD.

| **SPT parameters for detection and classification** | | | |
| --- | --- | --- | --- |
| **TrackMate** | | | |
| LoG estimated object diameter | 0.3 µm | LAP allows gap-closing events | Yes |
| LAP allows splitting and merging events | No | Maximum framte-to-frame linking distance | 0.5 µm |
| Maximum gap-closing distance | 0.5 µm | Maximum frame gap | 3 frames |
| **SPT script: options for trajectories and stall parameters** | | | |
| τ | 0.204 or 0.198 s | Moving window | 5 frames |
| v_t_ | 0.2 µm/s | Initial likelihood threshold (L_c1_) | 1.8 |
| Initial resident time threshold (t_c1_) | 5 frames | Secondary likelihood threshold (L_c2_) | 1.4 |
| Secondary resident time threshold (t_c2_) | 7 frames |  |  |
| **SPT script: limits for TAD analysis** | | | |
| D_block | 2*10^-3^ | γ_block | 0.4 |
| ν_block | 0.6 | γ_drift | 0.7 |
| ν_drift | 1.5 | lin_min & lin_max | 0.95 & 1.05 |
| l_mnw | 0.3 | l_mxw | 1.5 |
| dlin-coeff | 2.58 | γ-lim | 0.5 |
| D-lim | 1*10^-2^ |  |  |

**Tab. S1: Summary table of SPT script parameters for detection and classification.** Parameters divided into groups regarding TrackMate and SPT script described in the main text. In the software for MSS-TAD, the Alpha anomalous coefficient is called ν.

|  | **Blocked** | **Diffusive** | **Drifted** | **N** |
| --- | --- | --- | --- | --- |
| MIN6 ctrl | 47.8 ± 12.2 | 45.4 ± 11.1 | 6.8 ± 3.4 | 34 |
| MIN6 Nocodazole | 59.3 ± 12.7 | 36.3 ± 10.9 | 4.4 ± 3.3 | 38 |
| MIN6 Latrunculin-B | 27.7 ± 10.1 | 51.1 ± 10.5 | 21.2 ± 6.9 | 36 |

**Tab. S2 Summary table of the classified categories for MIN6.** Nomenclature abbreviations: Nocodazole (NCZ), Latrunculin-B (Lat-B), N is the numerosity of samples and all the data are expressed as length percentage with mean ± SD.


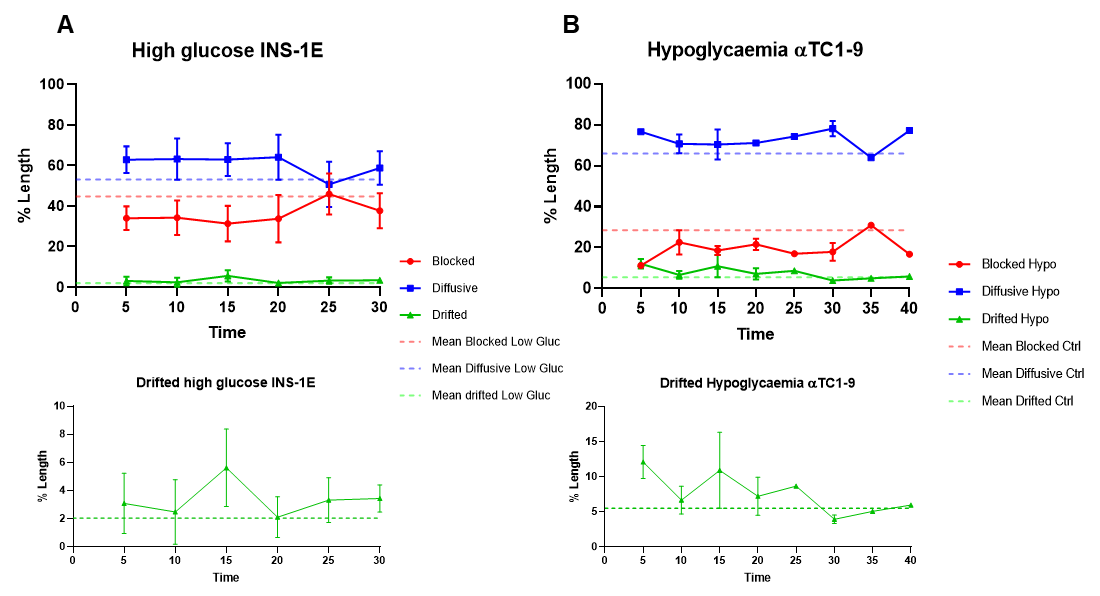


**Fig. S9 Time analysis of granule motility dynamics in INS-1E and αTC1-9 cells under stimulatory conditions.** Time points are considered as clusters of times; in detail: 5 stands for the first 0-5 minutes, 10 for 6-10 minutes, 15 for 11-15 minutes, 20 for 16-20, and so on. **A)** Compared to the control, the diffusive population is initially higher, likely indicating enhanced granule motility, but progressively decreases toward baseline over time. In contrast, the blocked population starts below control level and then gradually rises toward baseline. The drifted population shows a peak around 10–20 minutes, likely corresponding to an increase in directed transport that supports prolonged release once the RRP is depleted. **B)** At the initial time point for αTC1-9, both diffusive and drifted populations rise immediately after stimulation to facilitate glucagon secretion. In contrast, the blocked population remains below the control level. Compared to INS-1E β cells, αTC1-9 cells exhibit higher and more sustained granule motility that remains above control levels even after 40 minutes, following a distinct dynamic trend from INS-1E cells. About the drifted population we obtained a transient phase of increased dynamics in the initial phase of the secretion (0–25 minutes).


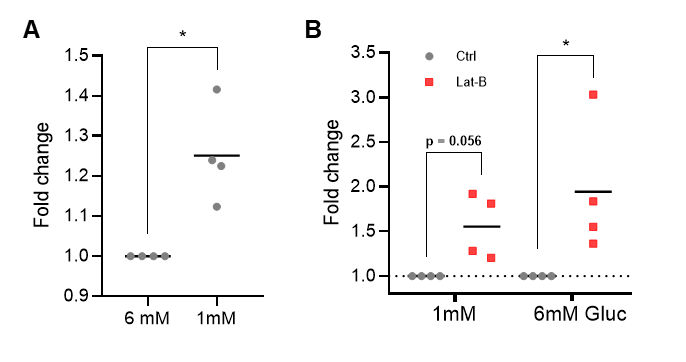


**Fig. S10 Glucagon secretion of αTC1-9 cells, expressed as fold change.** **(A)** Scatter plot showing normalized glucagon secretion under control (6 mM glucose) and hypoglycaemic (1 mM glucose) conditions (n = 4 biological replicates). A significant increase in secretion is observed at 1 mM glucose compared to the control condition. **(B)** Scatter plot illustrating normalized glucagon secretion in the presence or absence of Lat-B at the indicated glucose concentrations (Lat-B vs Ctrl, respectively). The data reveals a pro-secretory effect of Lat-B that appears to be independent of glucose concentration (n = 4 biological replicates).
